# miRNA-regulated delivery of lincRNA-p21 suppresses β-catenin signaling and tumorigenicity of colorectal cancer stem cells

**DOI:** 10.18632/oncotarget.5635

**Published:** 2015-10-16

**Authors:** Jun Wang, Zeng-jie Lei, Yan Guo, Tao Wang, Zhong-yi Qin, Hua-liang Xiao, Li-lin Fan, Dong-feng Chen, Xiu-wu Bian, Jia Liu, Bin Wang

**Affiliations:** ^1^ Department of Gastroenterology, Institute of Surgery Research, Daping Hospital, Third Military Medical University, Chongqing 400042, China; ^2^ Department of Pathology, Institute of Surgery Research, Daping Hospital, Third Military Medical University, Chongqing 400042, China; ^3^ Institute of Pathology and Southwest Cancer Center, Key Laboratory of Tumor Immunopathology of Ministry of Education of China, Southwest Hospital, Third Military Medical University, Chongqing 400038, China; ^4^ Institute of Translational Medicine, College of Medicine, Qingdao University, Qingdao 266021 China

**Keywords:** β-catenin signaling, colorectal cancer, cancer stem cells, LncRNA, miRNA

## Abstract

Cancer stem cells (CSCs) are key cellular targets for effective cancer therapy, due to their critical roles in cancer progression and chemo/radio-resistance. Emerging evidence demonstrates that long non-coding RNAs (lncRNAs) are important players in the biology of cancers. However, it remains unknown whether lncRNAs could be exploited to target CSCs. We report that large intergenic non-coding RNA p21 (lincRNA-p21) is a potent suppressor of stem-like traits of CSCs purified from both primary colorectal cancer (CRC) tissues and cell lines. A novel lincRNA-p21-expressing adenoviral vector, which was armed with miRNA responsive element (MRE) of miR-451 (Ad-lnc-p21-MRE), was generated to eliminate CRC CSCs. Integration of miR-451 MREs into the adenovirus efficiently delivered lincRNA-p21 into CSCs that contained low levels of miR-451. Moreover, lincRNA-p21 inhibited the activity of β-catenin signaling, thereby attenuating the viability, self-renewal, and glycolysis of CSCs *in vitro*. By limiting dilution and serial tumor formation assay, we demonstrated that Ad-lnc-p21-MRE significantly suppressed the self-renewal potential and tumorigenicity of CSCs in nude mice. Importantly, application of miR-451 MREs appeared to protect normal liver cells from off-target expression of lincRNA-p21 in both tumor-bearing and naïve mice. Taken together, these findings suggest that lncRNAs may be promising therapeutic molecules to eradicate CSCs and MREs of tumor-suppressor miRNAs, such as miR-451, may be exploited to ensure the specificity of CSC-targeting strategies.

## INTRODUCTION

Accumulating evidence demonstrates that cancer stem cells (CSCs) reside at the apex of tumor cell hierarchy and play crucial roles in growth of primary tumors and their metastases to distal organs [1–2]. CSCs exhibit potent capacity to evade immune surveillance by nature killer cells [3] and cytotoxic T cells [4], and intrinsic resistance to chemotherapeutic agents [5], thus constituting key targets for effective cancer therapy. The malignancy of colorectal cancer (CRC), the third most common cancer around the world, is tightly associated with a subset of CSCs. CRC CSCs are characterized by enhanced enzymatic activity of aldehyde dehydrogenase (ALDH) [6] and several other biomarkers [7–8]. The stem-like traits of CRC CSCs are maintained or acquired by the activation of several developmental pathways, especially the Wntless (Wnt)/β-catenin signaling [9]. In this regard, 90% of CRC contain a mutation in the adenomatous polyposis coli (APC) gene or other essential regulators of Wnt/β-catenin pathway [10]. In cells that have dysregulation of basal β-catenin activities, additional intrinsic or extrinsic oncogenic signals further contribute to the hyperactivation of Wnt/β-catenin pathway [11] and generation of CSC properties [10, 12]. Therefore, the regulatory network that fuels hyperactivation of β-catenin signaling is a reasonable target to eradicate CRC CSCs.

It is becoming increasingly clear that long non-coding RNAs (lncRNAs), non-protein-coding RNAs longer than 200 nucleotides, regulate various cellular functions and development processes. LncRNAs control gene expression by diverse modes, including epigenetic modification, microRNA (miRNA) sponging, and mRNA stabilization [13]. Consequently, aberrant expression of lncRNAs promotes tumorigenesis and metastasis of CRC and several other human cancers [14–15]. Importantly, lncRNA-mPvt1 enhances tumorsphere formation capacity of hepatocellular carcinoma cells [16]. This finding suggests that there may be other lncRNAs essential for the maintenance of CSC self-renewal. One of the candidates is the large intergenic noncoding RNA p21 (lincRNA-p21). LincRNA-p21 is a direct transcriptional target of p53 and reduces cell viability [17], suggesting its possible tumor suppressor function. Moreover, lincRNA-p21 selectively blocks the translation of β-catenin mRNAs, resulting in reduced levels of β-catenin protein in HeLa cells [18]. However, direct evidences are still lacking that support a role of lincRNA-p21 in CSC regulation.

A crucial challenge in eradicating CSCs is lack of suitable vehicles to specifically deliver therapeutic molecules into these cells while sparing normal tissues. One emerging strategy is to integrate miRNA response elements (MREs) into delivery vectors [19], since miRNAs that are significantly reduced in tumor cells and CSCs may allow for specific transgene expression in tumor tissues. For example, MREs of miRNA-150 specifically suppress gene expression in lymphocytes that contain high abundance of miRNA-150 and prevent transgene-induced lymphotoxicity [19]. Moreover, MREs of miR-34a, miR-137 and miR-182, whose expression levels are reduced in uveal melanoma cells, render specific introduction of Tumor Necrosis Factor-Related Apoptosis-Inducing Ligand (TRAIL) by adenovirus into tumor cells [20]. A recent study showed that expression of miR-451 was significantly downregulated in CRC CSCs, as compared to the bulk tumor cells [21]. Thus, we hypothesize that MREs of miR-451 may be utilized to specifically and efficiently deliver tumor suppressor lincRNAs into CSCs.

To this end, we assessed the potential role of lincRNA-p21 in regulating the stem-like traits of ALDH^+^ CRC CSCs. A recombinant adenoviral vector armed with MREs of miR-451 immediately following the lincRNA-p21-coding open reading frame (ORF) (Ad-lnc-p21-MRE) was constructed. The feasibility and effectiveness of Ad-lnc-p21-MRE for CSCs targeting was evaluated. Our results suggest that lincRNA-p21 is potentially applicable to eliminate CSCs and integration of miR-451 MREs significantly reduces its off-target expression in normal tissues.

## RESULTS

### The expression levels of lincRNA-p21 are reduced in ALDH^+^ CSCs and its restoration suppresses cancer stemness and tumorigenicity *in vitro*

**Figure 1:**
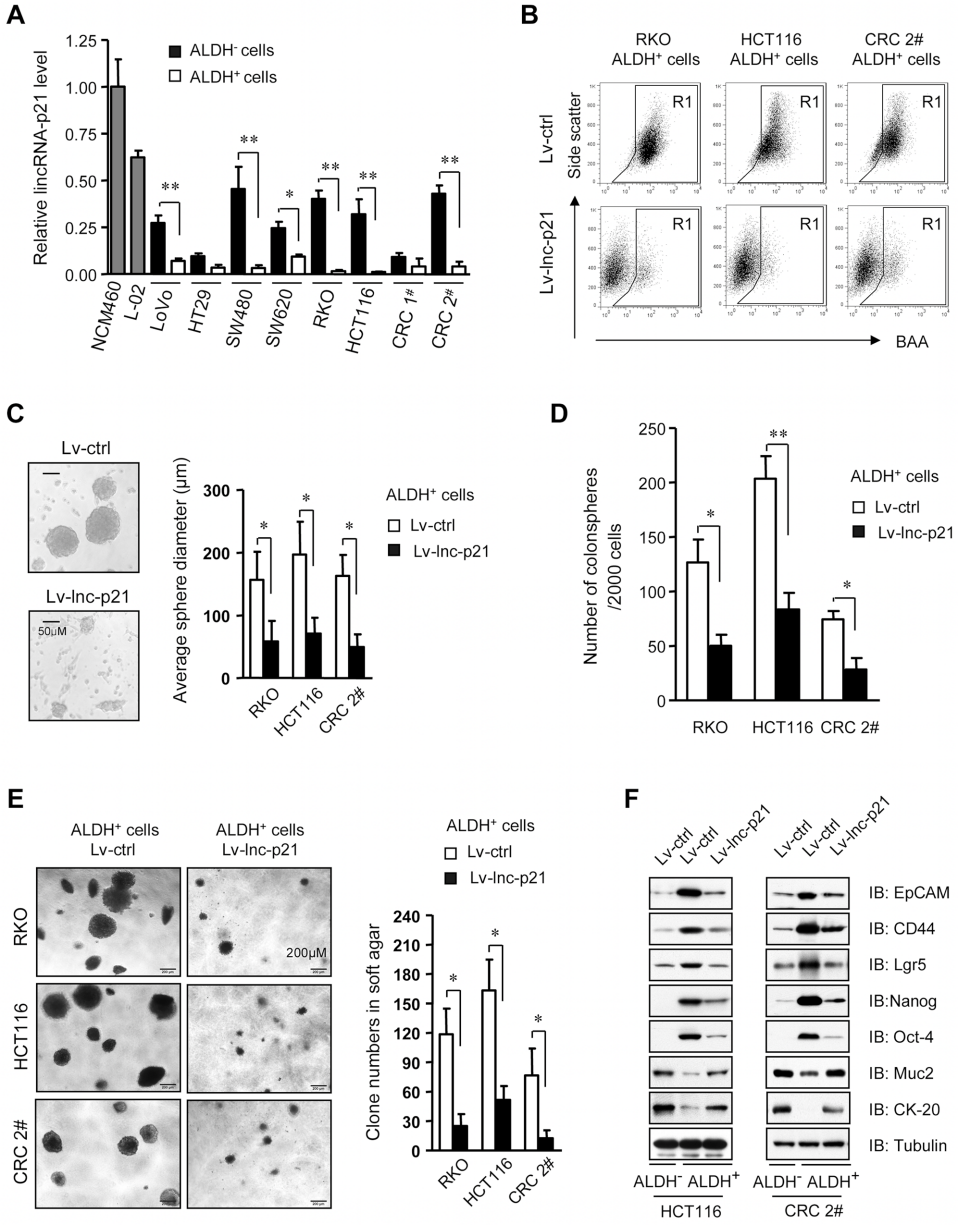
LincRNA-p21 represses stem-like properties and tumorigenicity of ALDH+ CSCs *in vitro* **A.** The levels of lincRNA-p21 were quantified by qPCR in ALDH^+^ CSCs and ALDH^−^ non-CSCs, as well as human immortalized normal colon epithelial cells (NCM460) and normal liver cells (L-02). *U6* was used as an endogenous control. CRC 1^#^ and 2^#^ represent two cases of primary CRC cells. **B.** Flow cytometric analysis of ALDH^+^ population in ALDH^+^ CSCs that were transduced with Lv-lincRNA-p21 (Lv-lnc-p21) or control lentiviral vector (Lv-ctrl) at 10 MOI for 7 days. **C, D.** ALDH^+^ CSCs were infected with Lv-lnc-p21 or Lv-ctrl (10 MOI). The diameter (C) and numbers of colonospheres (D) originating from single ALDH^+^ cells were measured with ImageJ software on Day 14. **E.** The numbers of soft agar colonies formed by these cells were counted and shown as means ± SD. Colonies with a diameter higher than 75 μm were counted. **F.** Stem cell markers, EpCAM, CD44, Lgr5, Nanog and Oct4, and differentiation markers Muc2 and CK-20 were examined by immunoblot analysis. Tubulin was a loading control. Representative graphs (B) or images (C, E, F) are shown. Data are presented as the mean ± SD (A, C, D, E) of each group from triple replicates. *P < 0.05, **P < 0.01.

We measured the expression level of lincRNA-p21 and found that it was significantly downregulated in CRC specimens, as compared to the paired non-cancerous colorectal epithelia from 24 patients (Supplementary Figure S1A). CRC CSCs were purified by fluorescence-activated cell sorting (FACS) based on ALDH activities of tumor cell subsets (Supplementary Figure S1B and S1C) [6, 22]. Importantly, the expression levels of lincRNA-p21 were significantly lower in ALDH^+^ CSCs than ALDH^−^ non-CSCs from several CRC cell lines as well as primary tumor cells CRC 1^#^ and 2^#^ (Figure 1A). The differential expression levels of lincRNA-p21 between CSCs and non-CSCs prompted us to investigate the potential roles of lincRNA-p21 in CRC CSCs.

We constructed a lentiviral vector carrying lincRNA-p21 (Lv-lnc-p21) to restore its expression in ALDH^+^ CSCs to the levels that were comparable to ALDH^−^ counterparts (Supplementary Figure S1D). Remarkably, the pool of ALDH^+^ cells was greatly reduced by Lv-lnc-p21 infection (Figure 1B). Analysis of tumorsphere formation, a hallmark of CSCs [6, 22], indicated that Lv-lnc-p21-infected ALDH^+^ CSCs formed smaller (Figure 1C) and less (Figure 1D) tumorspheres than those infected with control lentiviral vector (Lv-ctrl). Moreover, Lv-lnc-p21 infection reduced the growth of ALDH^+^ CSCs (Supplementary Figure S1E). Importantly, soft agar colony formation assays demonstrated that expression of exogenous lincRNA-p21 suppressed the tumorigenicity of single ALDH^+^ CSCs *in vitro* (Figure 1E).

Furthermore, we examined the expression levels of other putative CRC CSC markers, such as EpCAM [23], CD44 [22], and Lgr5 [24], pluripotency factors Nanog and Oct4, and differentiation markers of colorectal epithelium, Mucin2 and CK-20. We found that lincRNA-p21 partially inhibited the expression of stemness-associated markers while upregulated the levels of differentiation-associated genes (Figure 1F). Taken together, these data demonstrate that exogenous lincRNA-p21 significantly inhibits CSC function and tumorigenicity and induces partial differentiation of CRC CSC, suggesting the possibility of restoring lincRNA-p21 to eliminate CRC CSCs.

### Depletion of lincRNA-p21 confers on ALDH^−^ non-CSCs with stemness and tumorigenicity

**Figure 2:**
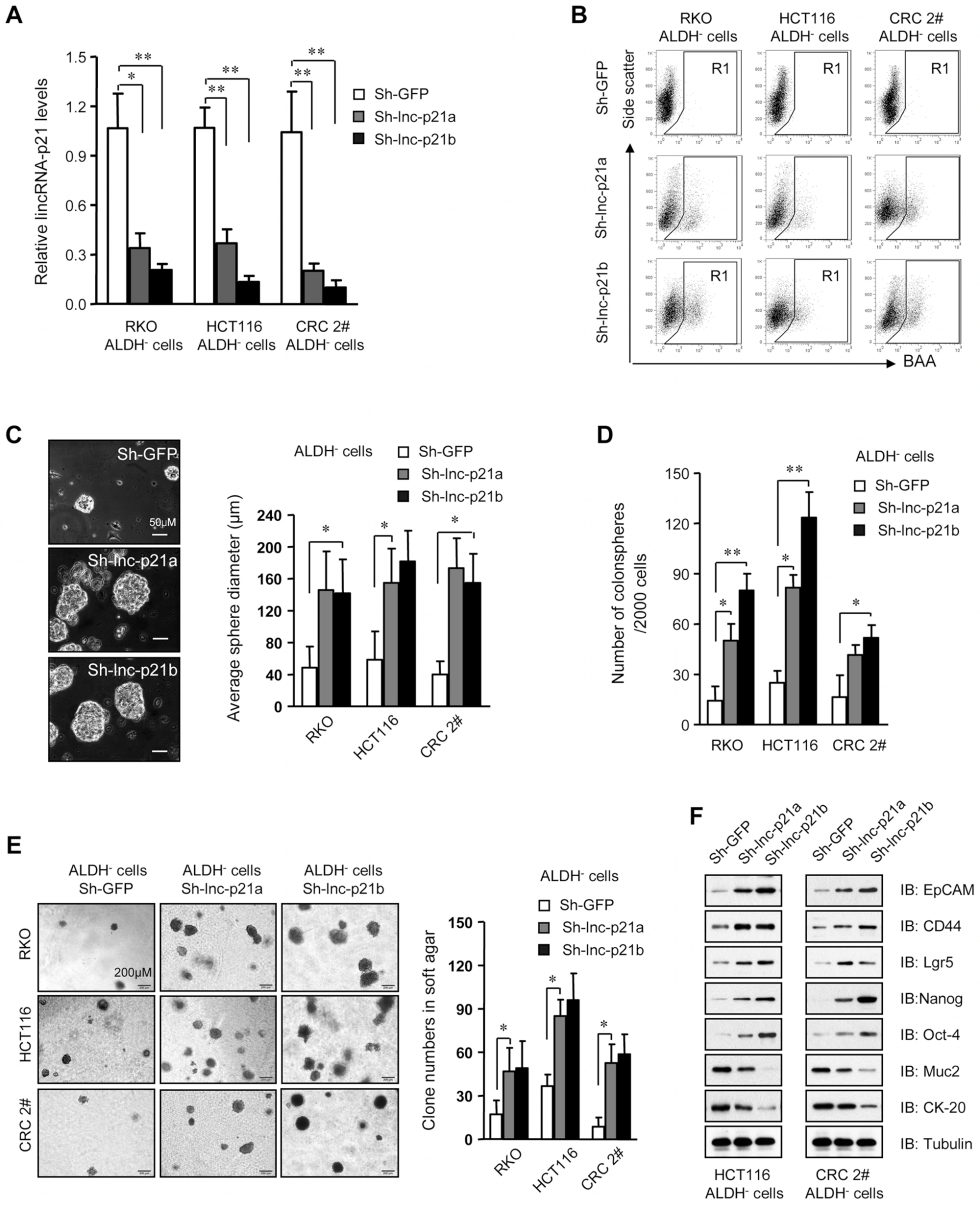
Knockdown of lincRNA-p21 enhances stemness and tumorigenicity of ALDH–CRC cells **A.** The level of lincRNA-p21 was evaluated by qPCR in ALDH^−^ cells infected with lentivirus expressing two independent lincRNA shRNAs, Sh-lnc-p21a and Sh-lnc-p21b, at 10 MOI for 48 hrs. Infection with shRNA targeting GFP, Sh-GFP, served as control. **B.** Flow cytometric analysis of ALDH^+^ population in ALDH^−^ cells after transduction for 7 days. **C, D.** The diameter (C) and numbers of spheres (D) generating by single ALDH^−^ cells were measured with ImageJ software 14 days after infection with lentiviruses. More than 10 repeat wells were counted for each group and spheres with a diameter larger than 50 μm were included. **E.** Numbers of colonies formed by ALDH^−^ cells with or without lincRNA-p21 knockdown in soft agar-containing medium. Colonies with a diameter higher than 75 μm were counted. **F.** Immunoblot analysis of stem cell markers and differentiation markers in ALDH^−^ cells infected with lincRNA shRNAs or sh-GFP. Tubulin was a loading control. Representative graphs (B) or images (C, E, F) are shown. Data are presented as the mean ± SD (A, C, D, E) of each group from triple replicates. *P < 0.05, **P < 0.01.

To further evaluate the role of lincRNA-p21 in the maintenance of CSC stemness, we employed lentiviral vectors that expressed two independent shRNAs targeting lincRNA-p21 (Sh-lnc-p21a and Sh-lnc-p21b) to knockdown endogenous lincRNA-p21 in ALDH^−^ CRC cells (Figure 2A). Interestingly, FACS analysis revealed that the ALDH^−^ cells were in part transformed to ALDH^+^ ones by Sh-lnc-p21-infection (Figure 2B), while these changes were not observed in Sh-GFP-infected cells, implying that loss of lincRNA-p21 may induce de-differentiation of ALDH^−^ cells to generate ALDH^+^ CSCs.

Moreover, knockdown of lincRNA-p21 promoted the growth of tumorspheres (Figure 2C) and increased the number of tumorspheres (Figure 2D) formed by ALDH^−^ non-CSCs in a dose-dependent manner in stem cell medium. Depletion of lincRNA-p21 also increased the proliferation rates of ALDH^−^ cells (Supplementary Figure S2A). Moreover, loss of lincRNA-p21 expression significantly was correlated with increased tumorigenicity of single ALDH^−^ cells *in vitro* (Figure 2E). Immunoblot assays demonstrated that silencing lincRNA-p21 by two independent shRNAs promoted the expression of stemness-associated biomarkers and suppressed differentiation-related genes (Figure 2F). Therefore, loss of lincRNA-p21 indeed confers stemness to ALDH^−^ non-CSCs and enhances their tumorigenicity, further demonstrating that low levels of endogenous lincRNA-p21 are critical for the maintenance of CRC CSCs.

### miR-451 expression is dramatically reduced in ALDH^+^ CSCs

**Figure 3:**
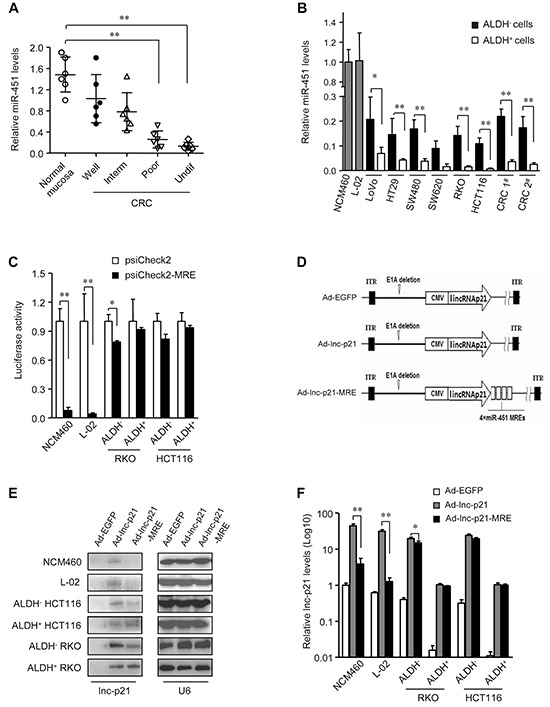
miR-451 MRE-regulated adenovirus specifically delivers lincRNA-p21 into CRC cells and CSCs **A.** The expression levels of miR-451 in normal mucosa and CRC samples of distinct grades of differentiation (Inter, intermediate; undif, undifferentiated) were measured by qPCR. U6 was used as an endogenous control. **B.** The levels of miR-451 were quantified by qPCR in normal cell lines and ALDH^+^ or ALDH^−^ subsets of CRC cells. U6 was used as endogenous control. **C.** Luciferase activity was detected in indicated types of cells after transfection with psiCheck (1 μg / 1 × 10^6^ cells) and psiCheck2-miR-451-MRE (1 μg / 1 × 10^6^ cells) for 48 hrs. **D.** Four copies of miR-451 MREs were inserted immediately following a lincRNA-p21-coding ORF on adenoviral vector Ad-lnc-p21 to generate Ad-nc-p21-MRE. Ad-EGFP served as control. **E.** The cells were infected with Ad-EGFP, Ad-lnc-p21, or Ad-lnc-p21-MRE at 10 MOI for 48 hrs. LincRNA-p21 expression was detected by Northern blotting. U6 was used as an endogenous control. **F.** LincRNA-p21 was further quantified by qPCR in these cells. U6 was a loading control. Representative images (E) are shown. Data are presented as the mean ± SD (A, B, C, F) of each group from triple replicates. *P < 0.05, **P < 0.01.

Several lines of evidence suggest that miR-451 acts as a tumor suppressor in multiple neoplasms [21, 25–27]. We also observed that miR-451 expression levels were significantly reduced in CRC tissues compared with normal colorectal epithelia, and inversely correlated with the grades of CRC tumors (Figure 3A). Moreover, expression levels of miR-451 were decreased in all examined colorectal cancer cells, as compared to NCM460 normal colon mucosal cells and L-02 normal hepatocytes (*P* < 0.01) (Figure 3B). Importantly, ALDH^+^ CSCs contained even lower levels of miR-451 than ALDH^−^ non-CSCs (Figure 3B). Furthermore, reduced expressions of miR-451 in ADLH^+^ CSCs were observed even after serial passages *in vitro*, as compared to the corresponding ALDH^−^ cells (Supplementary Figure S3A). The remarkable differences in the abundance of miR-451 between ALDH^+^ CSCs, ALDH^−^ non-CSCs and normal cells make its MRE an attractive modulator to ensure the CRC-specific expression of exogenous therapeutic genes.

### Application of miRNA response elements (MREs) of miR-451 prevents exogenous gene expression in normal cells

It is well known that disruption of the Wnt/β-catenin signaling attenuates the regenerative capacity of hepatic tissue [28]. Thus, off-target expression of lincRNA-p21 may raise the risk of severe side effects in future clinical application. To address this issue, four copies of miR-451 MREs were inserted into the psiCheck2 plasmid for luciferase expression (psiCheck2-MRE). Luciferase activities were greatly suppressed by more than 92% in miR-451-high-expressing NCM460 and L-02 cells that were transfected with psiCheck2-MRE (Figure 3C). In ALDH^−^ non-CSCs, the application of miR-451 MREs caused only a moderate reduction in luciferase expression (21.4% in ALDH^−^ HCT116 cells and 19.2% in ALDH^−^ RKO cells) (Figure 3C). Importantly, there was no significant difference in the luciferase activities between psiCheck2- or psiCheck2-MRE-transfected ALDH^+^ CSCs (Figure 3C). These data suggest that integration of miR-451 MREs does not significantly suppress the expression of exogenous gene in CSCs while sparing normal cells.

### miR-451 MRE-regulated adenovirus exhibits CRC specificity and facilitates efficient delivery of lincRNA-p21 into CSCs

Four MREs of miR-451 were incorporated into lincRNA-p21-expressing adenoviral vector (Ad-lnc-p21) to constrain its expression, which was designated as Ad-lnc-p21-MRE (Figure 3D). Transduction of Ad-lnc-p21 resulted in non-specific expression of lincRNA-p21 in normal NCM460 and L-02 cells, as well as in ALDH^+^ CSCs and ALDH^−^ non-CSCs. In contrast, infection of Ad-lnc-p21-MRE led to undetectable lincRNA-p21 expression in NCM460 and L-02 cells, and moderate expression level of lincRNA-p21 in ALDH^−^ CRC cells (Figure 3E). Intriguingly, Ad-lnc-p21-MRE-infected ALDH^+^ CSCs expressed comparable levels of lincRNA-p21 to those infected by Ad-lnc-p21 (Figure 3E). Further qPCR analyses confirmed that lincRNA-p21 expression was at comparable levels in ALDH^+^ cells infected with either Ad-lnc-p21 or Ad-lnc-p21-MRE (Figure 3F). Notably, Ad-lnc-p21-MRE spared normal cells but retained high expression of lincRNA-p21 in ALDH^+^ CSC (Figure 3F). These data demonstrate that integration of miR-451 MREs enables specific and efficient expression of lincRNA-p21 in ALDH^+^ CSCs.

### Ad-lnc-p21-MRE suppresses β-catenin signaling in ALDH^+^ CSCs in a miR-451-dependent manner

LincRNA-p21 was shown to reduce the levels of β-catenin protein in HeLa cells [18], indicating lincRNA-p21 could also inhibit β-catenin signaling in CRC cells. To this end, we observed that depletion of endogenous lincRNA-p21 in ALDH^−^ cells upregulated the abundance of β-catenin protein (Supplementary Figure S4A) and signaling activity (Supplementary Figure S4B) in a dose-dependent manner. Moreover, knockdown of lincRNA-p21 increased the mRNA levels of Lgr5 and Axin2, both of which are putative β-catenin target genes (Supplementary Figure S4C).

**Figure 4:**
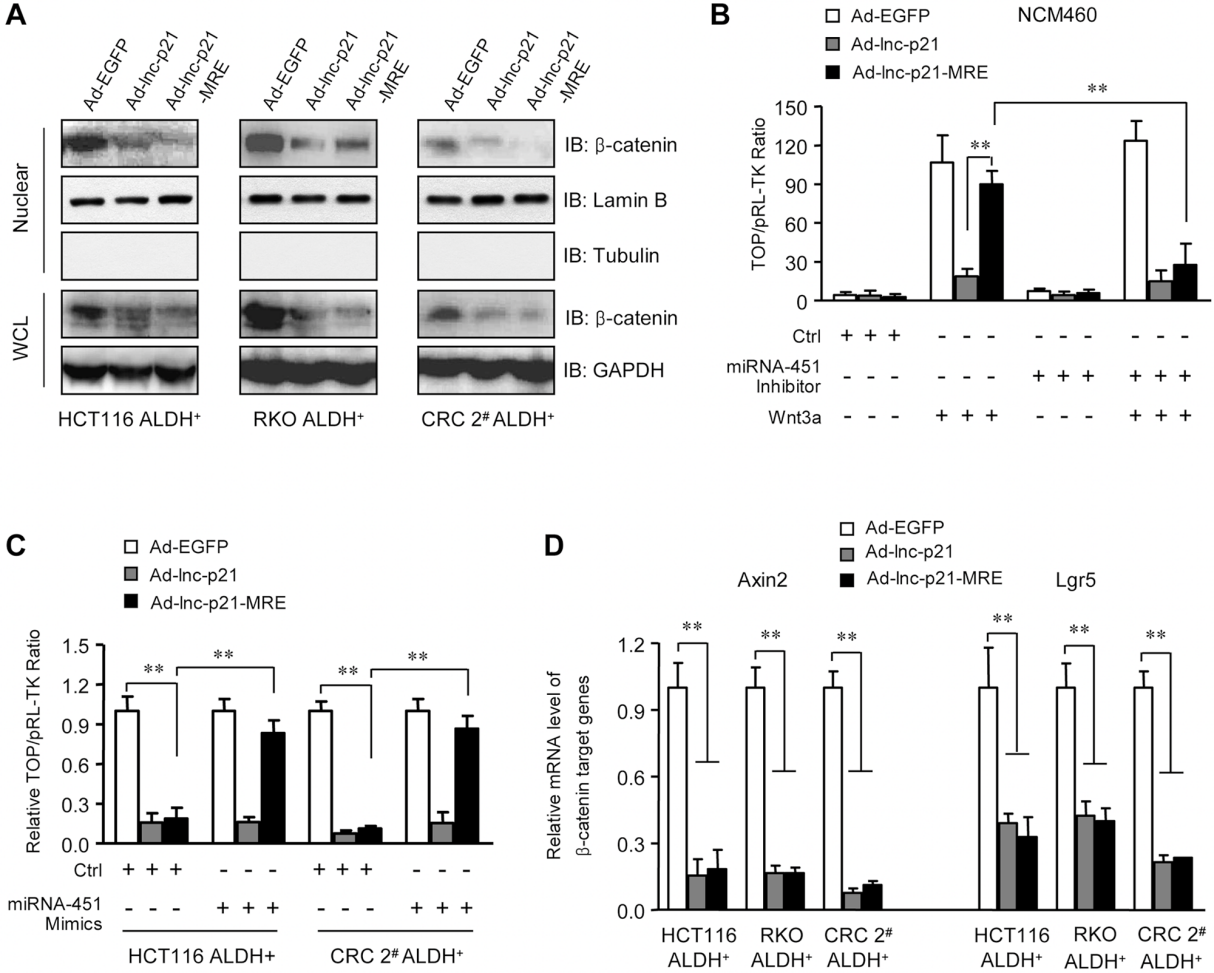
Ad-lnc-p21-MRE suppresses the activity of Wnt/β-catenin pathway in ALDH+ CSC in a miR-451-regulated manner **A.** ALDH^+^ CSCs were infected with 10 MOI of Ad-EGFP, Ad-lnc-p21 or Ad-lnc-p21-MRE. Cell lysate was collected 48 hrs later and the levels of β-catenin in nuclear extracts and whole cell lysate (WCL) were determined by western blot. Lamin B and GAPDH served as loading controls for nuclear lysate and WCL, respectively. **B.** NCM460 cells were transduced as in (A) The cells were co-transfected with the mixture of TOPFlash reporter plasmids and pRL-TK control plasmids (1 μg / 1 × 10^6^ cells; 40:1), and/or Wnt 3a-expressing plasmid (500 ng / 1 × 10^6^ cells) and miRNA-451 inhibitor (50 nM) for 48 hrs. TOPFlash reporter activity was examined to quantify the activity of β-catenin signaling. pRL-TK was used as a transfection control. **C.** ALDH^+^ CSCs were infected with indicated adenoviruses (10 MOI). The cells were co-transfected with the mixture of TOPFlash reporter plasmids and pRL-TK control plasmids (1 μg / 1 × 10^6^ cells; 40:1), with or without miRNA-451 inhibitor (50 nM). Forty eight hours later, TOPFlash reporter activity was determined. **D.** mRNA levels of β-catenin-responsive target genes, Axin2 and Lgr5, were detected in the ALDH^+^ CSCs with infections. GAPDH was used as an endogenous control. Representative images (A) are shown. Data are presented as the mean ± SD (C, D, E) of each group from triple replicates. **P < 0.01.

We next examined whether enforced expression of lincRNA-p21 could suppress the activity of β-catenin signaling pathway in the ALDH^+^ cells. Immunoblotting data showed that Ad-lnc-p21 and Ad-lnc-p21-MRE infections both resulted in decreases in the levels of β-catenin in nucleic and whole cell lysates of ALDH^+^ cells (Figure 4A). TOPFlash reporter assays confirmed the suppression of Wnt/β-catenin signaling by Ad-lnc-p21-MRE, which could be as efficient as Ad-lnc-p21, and was tightly controlled by endogenous miR-451 in cells. Specifically, inhibition of miR-451 facilitated efficient suppression of β-catenin signaling activity by Ad-lnc-p21-MRE in NCM460 cells (Figure 4B). Moreover, restoration of miR-451 expression rescued the activation of β-catenin signaling in ALDH^+^ CRC cells infected with Ad-lnc-p21-MRE (Figure 4C). Furthermore, exogenous lincRNA-p21 delivered by both Ad-lnc-p21 and Ad-lnc-p21-MRE downregulated putative β-catenin-responsive genes including Axin2 and Lgr5 (Figure 4D). Collectively, lincRNA-p21 overexpression by miR-451 MRE-regulated adenoviral vector suppresses the activation of β-catenin signaling in ALDH^+^ CSCs in a miR-451-dependent manner.

Furthermore, we examined the potential mechanisms by which overexpression of lincRNA-p21 reduces β-catenin protein in ALDH^+^ CSCs. Degradation of β-catenin protein is regulated by phosphoinositide-dependent kinase 1 (PDK1)/AKT/GSK-3β signaling pathway. We did not observed significant alterations in phosphorylation level and protein expression of AKT and GSK-3β (Supplementary Figure S4D). Moreover, ectopic expression of lincRNA-p21 did not down-regulate mRNA levels of β-catenin (Supplementary Figure S4E), suggesting that lincRNA-p21 may not inhibit β-catenin at transcriptional level. These findings imply that lincRNA-p21 overexpression may not suppress the activation of β-catenin by inducing protein degradation or transcriptional inhibition.

### Ad-lnc-p21-MRE reduces the cell viability and self-renewal of ALDH^+^ CSCs *via* attenuating β-catenin signaling *in vitro*

**Figure 5:**
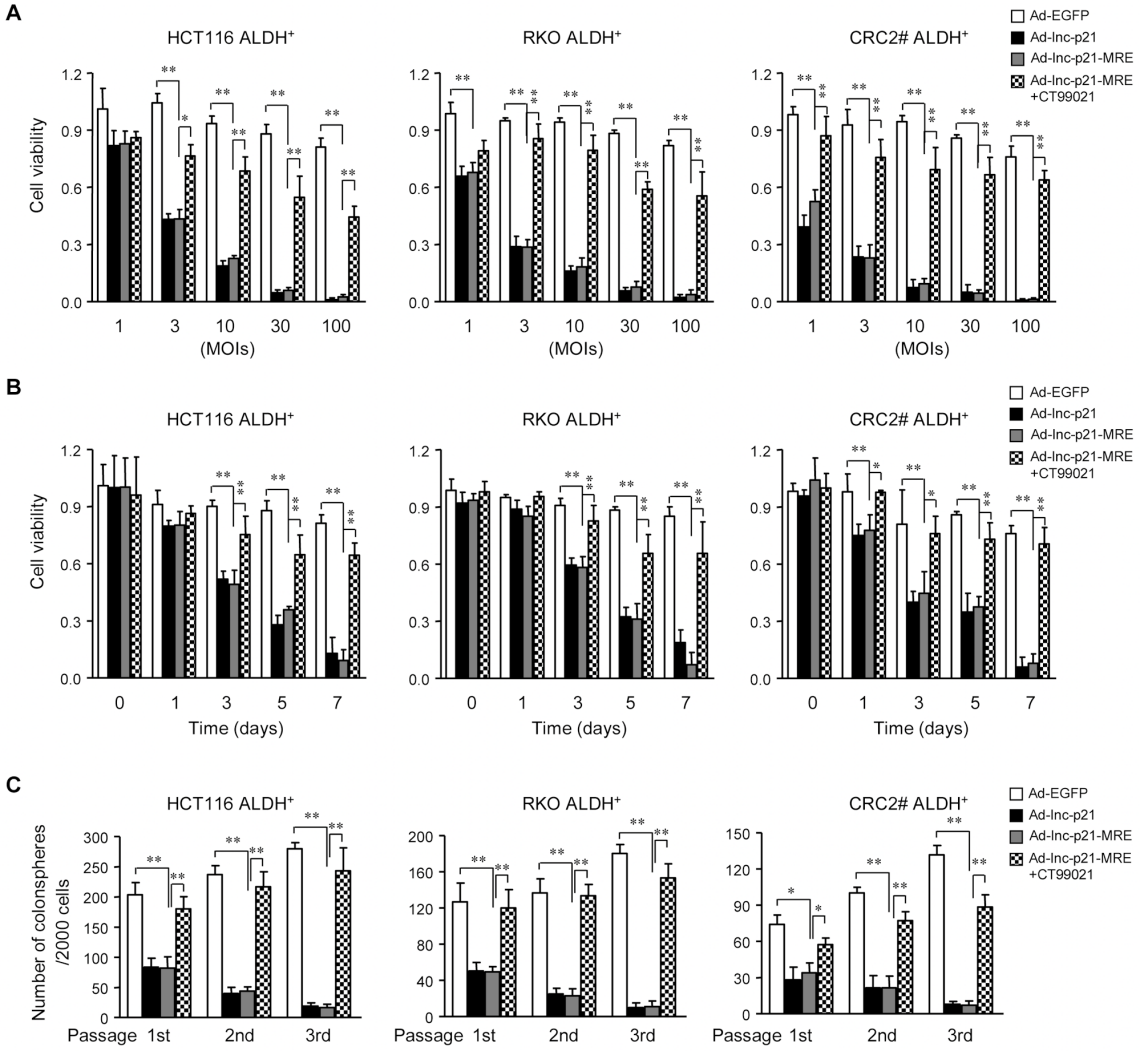
Ad-lnc-p21-MRE decreases the viability and stem-like traits of CSCs by suppressing β-catenin signaling **A.** ALDH^+^ CSCs were transduced with the adenoviruses at indicated MOIs for 7 days, or incubated together with a GSK-3 inhibitor CT99021 (3 μM). The cell viability was measured by CCK8 reagent. **B.** ALDH^+^ CSCs were infected with the adenoviral vectors (10 MOI), with or without CT99021 incubation (3 μM). The cell viability was determined by CCK8 assay kit at indicated time points. **C.** ALDH^+^ CSCs infected with the adenoviruses (10 MOIs) were cultured in stem cell medium for 14 days. CSCs infected with Ad-lnc-p21-MRE were further treated with CT99021(3 μM) or solvent. The numbers of spheres originating from single ALDH^+^ cells were quantified. The parental spheres were dissociated and secondary and tertiary sphere formation was also examined. All the data are presented as the mean ± SD of each group from triple replicates. *P < 0.05, **P < 0.01.

The Wnt/β-catenin signaling has been shown to be critical for self-renewal and propagation of CSC. Thus inhibition of β-catenin signaling by lincRNA-p21 overexpression may effectively target CSC. To this end, both Ad-lnc-p21 and Ad-lnc-p21-MRE exhibited comparable inhibition efficiencies on the viability of ALDH^+^ CSCs (Figure 5A). The inhibitory effect was dose-dependent, with 100 MOI of the adenoviruses showing a maximal suppression (approximately 90%) on cell viability (Figure 5A). The cytotoxicity of adenoviruses to ALDH^+^ CSCs also relied on the duration of incubation (Figure 5B). Moreover, CT99021, a specific GSK-3 inhibitor that significantly restored β-catenin protein levels (Supplementary Figure S5A) and signaling activity (Supplementary Figure S5B) in lincRNA-p21-overexpressing CSC, efficiently rescued the viability of ALDH^+^ CSCs infected with Ad-lnc-p21-MRE (Figure 5A and 5B). These data imply that both Ad-lnc-p21 and Ad-lnc-p21-MRE reduce the viability of ALDH^+^ CSCs at least partially by suppressing β-catenin signaling *in vitro*.

Next we analyzed the effects of adenovirus infection on tumorsphere formation by CSCs in a serial passage manner, which demonstrates their self-renewal capacity *in vitro* [22, 29]. Our data revealed that both Ad-lnc-p21 and Ad-lnc-p21-MRE infection significantly decreased the numbers of primary colonospheres derived from ALDH^+^ CSCs (Figure 5C). Moreover, infection of the adenoviruses also suppressed the generation of secondary and tertiary colonospheres. The reduction in colonosphere formation was efficiently attenuated by CT99021, which rescued the β-catenin pathway (Figure 5C and Supplementary Figure S5A). Additionally, we also observed that lincRNA-p21 overexpression induced elevated levels of cleaved caspase-3, a marker of caspase activation and cell apoptosis, in CSCs (Supplementary Figure S5A). Co-incubation with CT99021 inhibited the increases in caspase-3 activation (Supplementary Figure S5A), suggesting that inhibition of β-catenin activity by lincRNA-p21 overexpression in CSCs may also induce cellular apoptosis. Thus, delivery of exogenous lincRNA-p21 by adenoviruses inhibits propagation of CRC cells in part by eliminating self-renewing CSCs through inhibiting β-catenin signaling.

### Ad-lnc-p21-MRE inhibits β-catenin/PDK1 signaling axis to suppress aerobic glycolysis in ALDH^+^ CSCs

**Figure 6:**
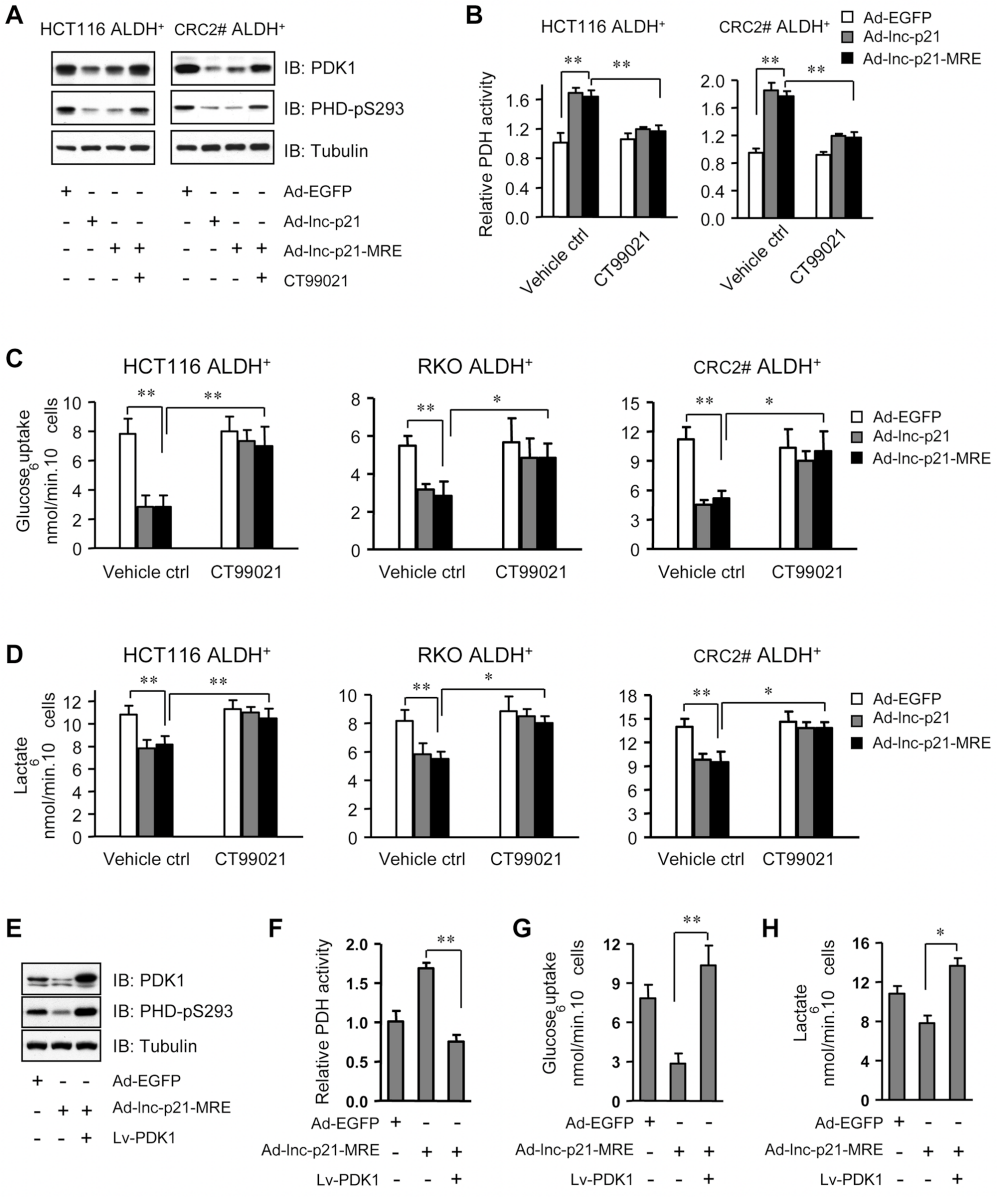
Ad-lnc-p21-MRE suppresses glycolysis of CSCs through downregulating β-catenin/PDK1 signaling axis **A.** The levels of pyruvate dehydrogenase kinase 1 (PDK1) and phosphorylated PDH (S293) were detected by immunoblot analysis in ALDH^+^ CSCs, 48 hrs after treatment with Ad-EGFP, Ad-lnc-p21, or Ad-lnc-p21-MRE (10 MOI), or Ad-lincRNA-p21-MRE infection together with CT99021 incubation (3 μM). **B.** Relative PDH activity was determined in ALDH^+^ CSCs, 48 hrs after infection with Ad-EGFP, Ad-lincRNA-p21 and Ad-lincRNA-p21-MRE (10 MOI). CT99021 (3 μM) was simultaneously used to rescue the inhibition of β-catenin signaling by lincRNA-p21. **C.** ALDH^+^ CSCs were treated as in (B) and their glucose consumption was determined by a colorimetric assay kit. **D.** Lactate production by these ALDH^+^ cells was assessed with accutrend lactate analyzer. **E.** ALDH^+^ CSCs isolated from HCT116 cells were infected with Ad-EGFP or Ad-lnc-p21-MRE (10 MOI), or co-infected with Ad-lincRNA-p21-MRE and Lv-PDK1 for 48 hrs. The levels of PDK1 and phosphorylated PDH (S293) were detected by Western blot. **F, G, H.** ALDH^+^ CSCs isolated from HCT116 cells were treated as in (E). Their relative PDH activity (F), glucose uptake (G) and lactate production (H) were measured. Representative images (A, E) are shown. Data are presented as the mean ± SD (B, C, D, F, G, H) of each group from triple replicates. *P < 0.05, **P < 0.01.

Since the β-catenin signaling was recently reported to switch oxidative phosphorylation to aerobic glycolysis by transcriptionally upregulating the expression of pyruvate dehydrogenase kinase 1 (PDK1) [30], it is conceivable that endogenous lincRNA-p21 might down-regulate PDK1 through inhibiting β-catenin expression in ALDH^+^ CSCs. To this end, depletion of endogenous lincRNA-p21 upregulated the levels of PDK1 mRNA (Supplementary Figure S6A) and protein (Supplementary Figure S6B) in a dose-dependent manner, which is associated with elevated phosphorylation of pyruvate dehydrogenase (PDH) at Serine 293, a direct substrate of PDK1 [31] (Supplementary Figure S6B). Consistently, lincRNA-p21 overexpression led to downregulated PDK1 mRNA (Supplementary Figure S6C) and protein expression (Figure 6A), and reduced Serine 293 phosphorylation of PDH (Figure 6A), and consequently increased PDH activity (Figure 6B). Interestingly, CT99021 treatment rescued the effect of lincRNA-p21 on PDK1 downregulation, PDH phosphorylation (Supplementary Figure S6C and Figure 6A) and activity changes (Figure 6B), which was likely due to restoration of β-catenin expression by GSK3-β inhibition (Supplementary Figure S5A).

This observation made us to hypothesize that lincRNA-p21 might inhibit glycolysis in ALDH^+^ CSCs. To this end, we observed that glucose uptake was suppressed by infection with Ad-lnc-p21- and Ad-lnc-p21-MRE (Figure 6C). Consistently, the levels of lactic acid, an end product of glycolysis, were also reduced by infection with Ad-lnc-p21 or Ad-lnc-p21-MRE (Figure 6D). Notably, stabilization of β-catenin by GSK3-β inhibition restored glucose uptake and lactate production in ALDH^+^ cells overexpressing lincRNA-p21 (Figure 6C and 6D), suggesting that Ad-lnc-p21 and Ad-lnc-p21-MRE attenuates glycolytic activity of ALDH^+^ CSCs in a β-catenin signaling-dependent mechanism.

To further dissect the role of PDK1 in mediating lincRNA-p21-suppressed glycolysis, we overexpressed PDK1 in ALDH^+^ cells overexpressing lincRNA-p21 (Figure 6E). We found that overexpression of PDK1 significantly upregulated Serine 293 phosphorylation of PDH (Figure 6E) to decrease PDH activity (Figure 6F), leading to restoration of glucose uptake (Figure 6G) and lactate production (Figure 6H) in ALDH^+^ cells overexpressing lincRNA-p21. However, overexpression of PDK1 only rescued the serial generation of colonospheres by ALDH^+^ cells overexpressing lincRNA-p21 to a lesser extent (Supplementary Figure S6D). Taken together, these findings suggest that the β-catenin/PDK1 signaling axis plays a critical role in lincRNA-p21-mediated suppression of glycolysis in ALDH^+^ CSCs.

### Ad-lnc-p21-MRE inhibits tumorigenicity by eliminating self-renewing ALDH^+^ CSC *in vivo*

**Figure 7:**
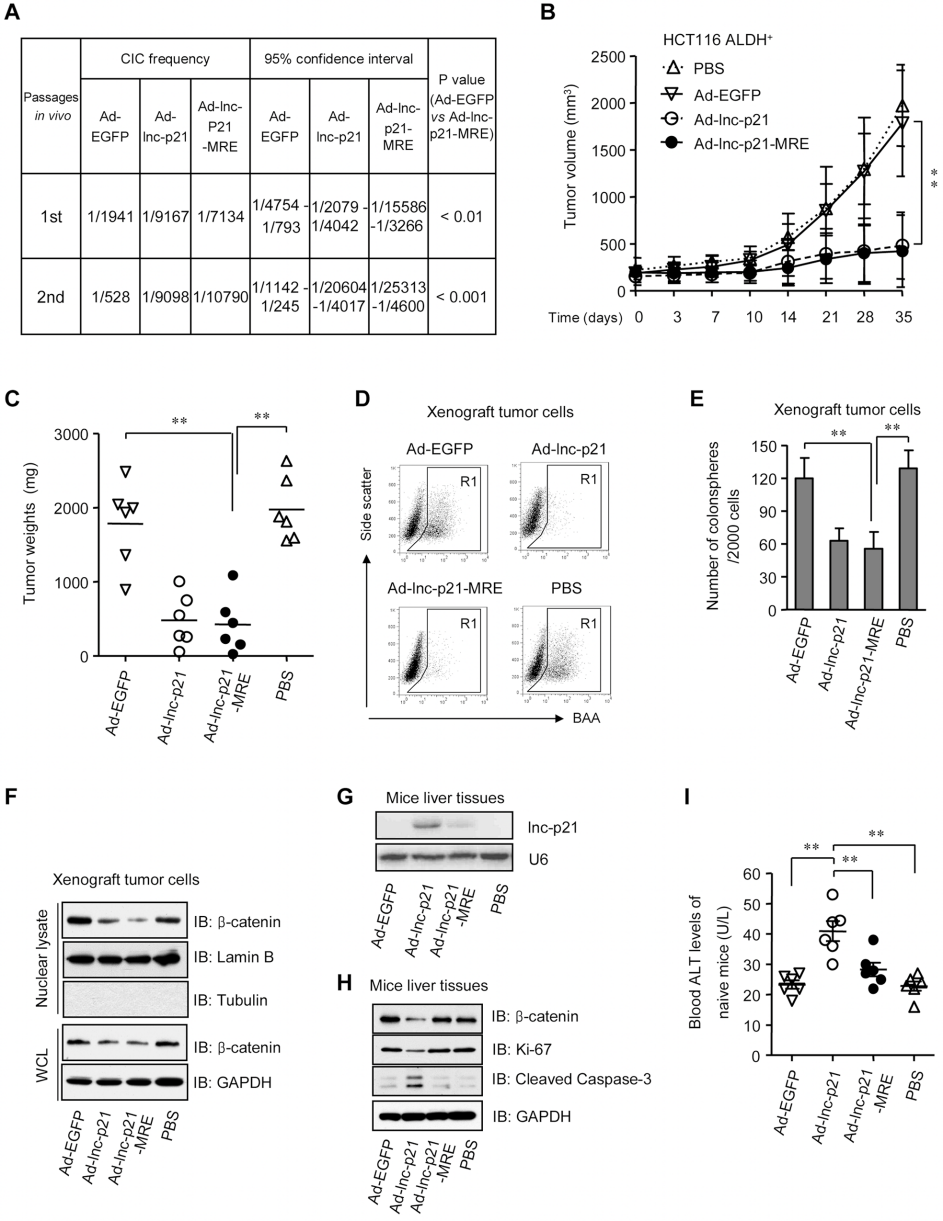
Ad-lnc-p21-MRE suppresses the growth and stemness of CSCs in nude mice and exhibits limited toxicities to normal tissues **A.** ALDH^+^ HCT116 cells were infected with either Ad-EGFP, Ad-lnc-p21, or Ad-lnc-p21-MRE and then transplanted subcutaneously in a limiting dilution manner in nude mice. The frequencies of cancer-initiating cells (CICs) were shown. Secondary tumor formation assays were performed to examine self-renewal potential *in vivo*. See also Supplementary Figure 7A. **B, C.** ALDH^+^ HCT116 cells (5 × 10^5^) was inoculated into the flanks of each male BALB/c nude mice (4 group; *n* = 6). The tumor-bearing mice were treated with ether Ad-EGFP, Ad-lnc-p21, or Ad-lnc-p21-MRE *via* tail vein injection. Control mice were treated with PBS. Growth curves (B) and weights (C) of the xenograft tumors were shown. **D, E.** Xenograft tumor cells were dissociated to make single cell suspension. The percentages of ALDH^+^ cells were examined by flow cytometry (D) Colonosphere formation assay were performed using single xenograft tumor cells and the numbers of tumorspheres were shown (E) **F.** The xenograft tumor cells were collected to examine the expression of β-catenin in nuclear lysate and whole cell lysate (WCL) by western blot. Lamin B and GAPDH served as loading controls for nuclear lysate and WCL, respectively. **G.** Human lincRNA-p21 expression was detected in mouse liver by Northern blotting. U6 was used as control. **H.** Western blot analyses of β-catenin, Ki-67, and cleaved caspase-3 expression in the liver tissues of recipient mice. **I.** Blood ALT levels of tumor-free healthy mice that were administrated *i.v.* with the adenoviruses or PBS (4 group; *n* = 6). Representative graphs (D) or images (F, G, H) are shown. Data are presented as the mean ± SD (B, C, E, I) of each group from triple replicates. *P < 0.05, **P < 0.01.

To evaluate the effects of adenovirus on the self-renewal and tumorigenicity of CSCs *in vivo*, we first performed limiting dilution assays in a serial transplantation manner in nude mice using HCT116 ALDH^+^ cells infected with Ad-EGFP, Ad-lnc-p21 or Ad-lnc-p21-MRE. Compared to Ad-EGFP, both Ad-lnc-p21-MRE and Ad-lnc-p21 showed a similar capacity to inhibit the formation of xenografts by CSCs (Supplementary Figure S7A), resulting in significant decreases in the frequencies of cancer-initiating cells (CICs) (Figure 7A). In a secondary transplantation assay, a golden standard of assessing the self-renewal of CSCs *in vivo*, treatment with Ad-lnc-p21-MRE and Ad-lnc-p21 led to 20-fold and 17-fold reductions in the frequencies of CICs, respectively (Figure 7A), supporting the notion that Ad-lnc-p21-MRE infection efficiently reduced self-renewal capacity of CSC *in vivo*.

To further examine the physiological role of Ad-lnc-p21-MRE in suppressing ALDH^+^ CSC function, ALDH^+^ CSCs isolated from HCT116 cells were subcutaneously inoculated in the flanks of nude mice. After tumor establishment, the mice were then systemically administrated with adenoviruses Ad-EGFP, Ad-lnc-p21 or Ad-lnc-p21-MRE, and the growth of xenograft tumors was examined. Thirty-five days after the first administration of adenoviruses, Ad-lnc-p21-MRE suppressed the size and weight of CSC xenotransplants by 74.6% and 72.8%, respectively (Figure 7B and 7C). Its inhibitory effect was similar to that of Ad-lnc-p21 (70.7% and 71.5%, respectively), while no significant suppression effect was observed in the Ad-EGFP-treated mice.

Moreover, analysis of the dissected tumors revealed that treatment with Ad-lnc-p21-MRE and Ad-lnc-p21 decreased the pool of ALDH^+^ population (Figure 7D) and the ability of xenograft cancer cells to form colonospheres (Figure 7E). Furthermore, the activation of β-catenin pathway was suppressed by overexpression of lincRNA-p21, as evidenced by the reduction in the expression levels of β-catenin in both nuclear and whole cell lysates (Figure 7F). Therefore, *in vivo* treatment with Ad-lnc-p21-MRE potently inhibits the self-renewal of CSCs, thereby restraining CRC tumorigenicity.

### Incorporation of miR-451 MREs minimizes the off-target expression of lincRNA-p21 and its toxicities to normal hepatocytes

Given that both Ad-lnc-p21 and Ad-lnc-p21-MRE exert similar effects on shrinking tumors, as well as concerning that systemic delivery of lincRNA-p21 may have potential influence on liver cell function, the level of human lincRNA-p21 in liver tissues of recipient mice were examined by Northern blotting. We found that Ad-lnc-p21 administration led to high expression of lincRNA-p21 in mouse livers while MRE-regulated vector had no significant off-target expression (Figure 7G). Moreover, Ad-lnc-p21 therapy inhibited the levels of β-catenin and Ki-67, while upregulated the expression of cleaved caspase-3, in both liver tissue (Figure 7H) and colon mucosa (Supplementary Figure S7B) of recipient tumor-bearing mice, although no significant differences in body weights were observed between Ad-lnc-p21 and Ad-lnc-p21-MRE groups (Supplementary Figure S7C). These data imply that application of miR-451 MRE may prevent lincRNA-p21 expression in the normal tissues of the mice, thus reducing side-effects such as growth inhibition and apoptosis induction towards healthy cells.

To further evaluate potential side effects of lincRNA-p21 delivery, we used a group of tumor-free mice to study the effects imposed by the constructs on healthy animals. Although systemic administration of Ad-EGFP, Ad-lnc-p21, or Ad-lnc-p21-MRE did not result in significant loss in body weights (Supplementary Figure S7D), the level of alanine aminotransferase (ALT), an useful indicator of liver cell injury, was significantly higher in the peripheral blood of mice inoculated with Ad-lnc-p21, as compared to controls (Figure 7I). Importantly, injection with Ad-lnc-p21-MRE did not elevate blood ALT levels in recipient mice (Figure 7I), suggesting that integration of miRNA-451 MRE in the adenovirus at least protected the naïve mice from hepatocyte damage induced by lincRNA-p21 overexpression.

## DISCUSSION

The precise molecular mechanisms underlying the self-renewing capacity of CSCs remain largely unclear [2]. We herein uncover a novel role of lincRNA-p21 in repression of stem-like phenotypes in CRC cells. Previous studies have demonstrated that lincRNA-p21 expression is induced by p53 [17] and could directly downregulate the levels of β-catenin protein in cervical carcinoma HeLa cells [18], implying that it may be a potential suppressor of tumorigenesis. However, stabilization of HIF-1α by lincRNA-p21 under hypoxia conditions implies that it could also play an oncogenic role [32]. We found that lincRNA-p21 expression levels were significantly reduced in CRC tissues, which are consistent with data from recent reports [33–34]. Thus, the function of lincRNA-p21 in regulating tumorigenesis is most likely context-dependent. More importantly, CSCs isolated from both primary CRC tissues and cell lines contain very low abundance of lincRNA-p21 and its restoration potently suppresses cancer stemness and tumorigenicity. Furthermore, loss-of-function studies directly demonstrated that endogenous lincRNA-p21 represses de-differentiation and prevents the acquisition of stemness and tumorigenicity of cancer cells. It is interesting that lincRNA-p21 has been reported to impair somatic cell reprogramming [35]. Therefore, the revelation of lincRNA-p21 as a potent suppressor on the stem-like properties of CRC cells suggests lincRNA-p21 as a valuable molecule for gene therapy against CSCs.

We demonstrated that MRE of tumor suppressor miRNAs could be exploited to enable specific transgene expression in CSCs and that miR-451 was such a candidate for CRC. miR-451 is downregulated in gastric cancer [26], breast cancer, and head and neck squamous cell carcinoma [27], where it executes tumor suppressor functions by targeting macrophage migration inhibitory factors and a disintegrin and metalloproteinase (ADAM) protein family members ADAMTS5 and ADAM10. Additionally, the levels of miR-451 are reduced in cancer cells to facilitate both chemo- and radio- resistance [25, 36]. Furthermore, miR-451 negatively regulates self-renewal, tumorigenicity, and chemoresistance of colorectal CSCs [21]. Here, we observed that miR-451 levels were significantly reduced in CRC cells. In addition, ALDH^+^ CSCs exhibited much lower expression of miR-451 than ALDH^−^ cells. Thus, we integrated MREs of miR-451 into the lincRNA-p21-expressing adenovirus and low levels of miR-451 in CRC cells, especially ALDH^+^ CSCs, allowed efficient introduction of lincRNA-p21, while high abundance of miR-451 avoided transgene expression in normal colon mucosal and liver cells *in vitro* and *in vivo*. Previous studies demonstrated that other miRNAs, such as let-7 [37], miR-100, and miR-200c [38], were also downregulated in CSCs. Our results imply that MREs of these miRNAs might also facilitate the selective delivery of therapeutic genes in CSCs. Moreover, we recently demonstrated that oncogenic miRNAs, such as miRNA-20a and miRNA-106a, were critical targets for inhibiting malignant phenotypes of CSCs [3, 39]. Thus, engineering MREs of tumor suppressor miRNAs represents an alternative approach by which CSCs-associated miRNAs could be utilized for targeted therapy.

miR-451 MRE-mediated delivery of lincRNA-p21 by adenovirus effectively suppresses malignant phenotypes of CRC CSCs in a cancer-specific fashion. Infection with either Ad-lnc-p21 or Ad-lnc-p21-MRE significantly inhibited the viability and self-renewal capacity of ALDH^+^ CSCs *in vitro* and *in vivo*. Importantly, Ad-lnc-p21-MRE therapy potently shrunk the CSC pool in nude mice. The therapeutic effects of the adenovirus appeared to be dependent on the inhibition of β-catenin signaling activity, as co-incubation with CT99021, a GSK-3 inhibitor to restore β-catenin activity, rescued the viability and stemness of ALDH^+^ CSCs expressing lincRNA-p21 (Supplementary Figure S7E). We have previously reported that Dickkopf-1 overexpression by a chimeric 5/35 adenovirus attenuates Wnt signaling and suppresses tumorigenicity of gastric CSCs [40]. These results, together with the observations that the Wnt/β-catenin pathway is essential for de-differentiation-mediated generation of CSCs [9], highlight β-catenin signaling as an important target for CSC therapy. More importantly, Ad-lnc-p21-MRE exhibits comparable inhibition efficiency on malignant phenotypes of CSCs, as compared with Ad-lnc-p21. However, Ad-lnc-p21-MRE infection does not result in expression of exogenous gene in normal cells, highlighting the safety of this engineered adenoviral vector for gene therapy.

Furthermore, Ad-lnc-p21-MRE efficiently inhibits aerobic glycolysis of ALDH^+^ CSCs, suggesting that metabolic alteration in CSCs may be targeted to reverse their malignant phenotypes. There are emerging evidences that CSCs in several tissues such as non-small cell lung cancer [41], pancreatic and ovarian cancers [42], glioma [42–43], and breast cancers [44] display elevated glycolytic metabolism, as compared to their differentiated counterparts. Intriguingly, reversal of glycolysis to oxidative phosphorylation is associated with impairment of propagation and stem cell traits of CSCs [41, 43–45]. By using Ad-lnc-p21-MRE, we present an effective approach to switch the program of glucose metabolism in CSCs, which may help eliminate these cells in cancer tissues. Inhibition of glycolysis in ALDH^+^ CSCs by Ad-lnc-p21-MRE is dependent on the inactivation of the β-catenin signaling, which regulates the PDK1/PDH signal axis to shut down mitochondrial oxidative phosphorylation (Supplementary Figure S7E) [30]. Since the Wnt signaling pathway is an essential pathway for CSC maintenance in many cancers [1–2, 11, 46], further investigation is needed to test whether Ad-lnc-p21-MRE could inhibit glucose metabolism in a broader range of scenarios.

Our current vector system is generated based on a replication-incompetent adenovirus. Therefore, Ad-lnc-p21-MRE is lack of proliferation capacity in the infected cells. This weakness may compromise the anti-tumor potency of therapeutic genes. Conditionally replicative adenoviral vectors, which retain their replication ability in cancer cells [47], may be selected as vectors to express tumor suppressor genes to a higher level. Additionally, incorporation of promoters that specifically exhibit high transcriptional activity in CSC may further facilitate the selective replication of these adenoviruses in the cell types of interest.

Collectively, we constructed a novel adenoviral vector that expressed lincRNA-p21 in a miR-451-regulated fashion. This vector delivered lincRNA-p21 gene preferentially in CRC CSCs and blocked the activation of Wnt/β-catenin signaling. The viability, self-renewal, and tumorigenicity of CSCs were greatly compromised by lincRNA-p21 overexpression. We provide evidence that MRE-regulated gene therapy is promising for CSC-targeting strategy and lncRNAs, such as lincRNA-p21, may be potential therapeutic genes against CSCs.

## MATERIALS AND METHODS

### Cell culture

Human colorectal cell lines (SW480, SW620, HCT116, LoVo, HT29 and RKO) were purchased from American Type Culture Collection (Manassas, VA). Immortalized normal colon epithelial cells (NCM460) and human normal liver cells (L-02) were obtained from Shanghai Cell Collection (Shanghai, China). HEK-293 human embryonic kidney cell line was obtained from Microbix Biosystems (Toronto, Canada). The cells were cultured in recommended media supplemented with 4 mM glutamine, 100 units/ml penicillin, and 100 μg/ml streptomycin in a 5% CO_2_ and humidified atmosphere at 37°C.

CRC tissues were obtained with written consent from patients undergoing surgical treatment at the Department of General Surgery in the Institute of Surgery Research, Daping Hospital. Primary culture of tumor cells were performed according to our standard protocol described previously [3, 39]. The patient-derived tumor cells were maintained in stem cell medium (DMEM/F12 supplemented with 10 ng/mL bFGF (PeproTech Asia), 20 ng/mL EGF (PeproTech Asia) and 10 ng/mL HGF (Peprotech), B27 (GIBCO), N2 (GIBCO), lipids (Sigma), heparin (4 μg/mL), and 1% penicillin-streptomycin) for expansion and cell sorting [21, 29].

### Fluorescence activated cell sorting (FACS)

Primary CRC cells and cell lines were fractionated for ALDH^+^ and ALDH^−^ subpopulations by fluorescence activated cell sorting (FACS) using an ALDEFLUOR assay kit (Stem Cell Technologies, Canada). The cells were collected and incubated with ALDH substrate in assay buffer for 45 min at 37°C. For negative control, cells were incubated with an ALDH inhibitor, diethylamino-benzaldehyde (DEAB). After incubation, the cells were then suspended in assay buffer for sorting on a BDAria II sorter (BD Biosciences, CA). ALDH^+^ CRC cells were maintained as colonospheres in stem cell medium [21, 29] on 6-well low-attachment plates at 3.5 × 10^5^/well for further analysis.

### Quantitative PCR (qPCR)

Total RNA was extracted using Trizol solution (Sigma-Aldrich, MO). Reverse transcription reaction was performed with TaqMan®MicroRNA Reverse Transcription Kit (Applied Biosystems). To detect *miR-451* expression level, qPCR was performed using TaqMan® 2 × Universal PCR Master Mix (Applied Biosystems) on CFX96™ Real-Time PCR Detection System (Bio-Rad Laboratories, CA). To detect the abundance of lincRNA-p21 transcript, cells were seeded in each well of 6-well plates overnight and infected with either Ad-EGFP, or Ad-lnc-p21-MRE, or Ad-lnc-p21 at 10 MOI. Total RNA were extracted 48 hrs later and transcribed into cDNAs using Rever Tra Ace qPCR RT Kit (Toyobo, Japan). The primer sequences for β-catenin targets were described previously [40]. Forward primer sequences for lincRNA-p21 is 5′-TGTTGCATTGTTGCATCATC-3′ and reverse primer is 5′-TTTCTTCCAGTGGTGAGTGG-3′.

### Northern blotting

Northern blotting assay was employed to detect the expression level of lncRNA-p21 in different cell lines with or without infection of the adenoviruses. The procedures were described in previous publications [20, 48].

### Cell viability assay

The viability of cancer cells was measured using CCK8 assay kit (Dojindo, Japan). Briefly, cells at a density of 5 × 10^2^ cells/well were plated in 96-well plates overnight and then infected with indicated adenoviruses or co-incubated with GSK-3 inhibitor CT99021 (3 μM) for indicated duration. Subsequently, 10 μl of CCK8 reagent (100 μl medium/well) was added to the culture medium for 1 h at 37°C, 5% CO_2_. The absorbance of optical density was determined using Varioskan Flash (Thermo Scientific, USA) at 450 nm (A450). Data were expressed as the percentage of viability as following: relative cell viability= [A450(treated)-A450(blank)] /[A450(control)-A450(blank)] ×100%.

### Luciferase reporter assay

Paired primers containing 4 copies of MREs of *miR-451* are as following: Forward: 5′-TCGAGGATATCACAAACACCAACGGTTAAC AAA CACCAACGGTTAACAAA CACCAACGGTTAACAAA CACCAACGGTTAACAAACACCGATATCGC-3′; Reve- rse:5′-GGCCGCGATATCGGTGTTTGTTAACCGTTGG TGTTTGTTAACCGTTGGTGTTTGTTAACCGTTGGT GTTTGTTAACCGTTGGTGTTTGTGATATCC-3′. (MRE sequences were underscored). The primers were annealed and inserted into psiCheck2 (Promega, WI) at the site of *XhoI* and *NotI* to construct MRE-regulated luciferase reporter plasmids psiCheck2-MRE. Cells were plated at 5 × 10^4^/well in 24-well plate and transfected with 200 ng of psiCheck2 or psiCheck2-MREs using Lipofectamine 2000 (Invitrogen). Forty eight hrs later, cell lysate was collected to measure Firefly and Renilla luciferase activities using the Dual-Luciferase reporter system (Promega, WI) [49].

### Package of adenovirus and lentivirus constructs

Ad-lnc-p21 was constructed based on a chimeric 5/35 adenovirus used in our previous study [40]. The partial sequence of lincRNA-p21 was obtained by PCR using cDNA derived from HEK-293 cells (The sequence is identical to a previous report [18]). The fragment was inserted into pShuttle-CMV to generate pShuttle-CMV-lincRNA-p21. pShuttle-CMV-lincRNA-p21 and pAdEasy-35 that contains 5/35 chimeric fiber were then co-transfected into *E. coli* BJ5183 to obtain recombinant plasmid pAd-lincRNA-p21. pAd-lincRNA-p21 was subsequently transfected into HEK-293 cells using the Lipofectamine 2000 (Invitrogen). After plague purification for three times and identification by PCR, recombinant adenovirus Ad-lnc-p21 was harvested and purified using CsCl gradient centrifugation. The titers of the adenovirus were quantified with TCID_50_ method using HEK-293 cells.

To generate Ad-lnc-p21-MRE, the DNA fragment containing 4 copies of MREs of miR-451 was inserted into pShuttle-CMV-lincRNA-p21 to generate pShuttle-CMV-lincRNA-p21-MRE and then package Ad-lnc-p21-MRE. To generate lentiviral vector expressing lincRNA-p21, the abovementioned partial sequence of lincRNA-p21 was inserted into pLVX-IRES-ZsGreen1 to generate pLVX-lincRNA-p21-IRES-ZsGreen1, followed by co-transfection with pSpax2 and pMD2G into HEK-293T cells. The lentivirus-containing broth was harvested 48 and 72 h after the transfection. The lentiviral vector was designated as Lv-lnc-p21.

For the construction of Sh-lincRNA-p21a and Sh-lincRNA-p21b, two different shRNA sequences (a: CTGCAAGGCCGCATGATGA; b: TGA AAAGAGCCGUGAGCUA) were inserted into pLK0.1-cloning vector, followed by lentivirus package in HEK-293T cells. The lentiviral vectors were designated as Sh-lnc-p21a and Sh-lnc-p21b, respectively. The full length cDNA of human PDK1 gene was also inserted into pLK0.1-cloning vector to generate Lenti-PDK1 expression virus (Lv-PDK1).

### Immunoblotting assay

Whole cell lysate was prepared using M-PER® Mammalian Protein Extraction Reagent (Thermo Scientific, IL). Nuclear extracts were collected using Active Motif nuclear extract kit. Protein concentration was determined using a BCA protein assay kit (Thermo Scientific, Pierce, Rockford, IL). Extracts were separated by SDS-PAGE and transferred onto polyvinylidene difluoride membranes (Millipore). After being blocked with 5% fat-free dry milk for 1 hrs, the membranes were incubated with primary antibodies at 4°C (β-catenin, 1:2000; PDK1, 1:2000; pPDH (S293), 1:2000; GAPDH, 1:2000; β-tubulin, 1:2000; Lamin B, 1:2000; GSK-3β, 1:1000; AKT-pT308, 1:1000; AKT-total, 1:1000). All the antibodies were purchased from Cell Signaling Technology). Overnight, the membranes were incubated with secondary antibodies for 1 hrs and visualized with SuperSignal West Dura Extended Duration Substrate (Thermo Scientific, IL).

### TCF reporter assay

The activity of Wnt signaling was examined by TCF reporter dual luciferase assay [40, 50]. Cells were transfected with TCF reporter vector (TOPflash) (Millipore, Billercia, MA, USA) and control renilla luciferase reporter vector (pRL-TK) (40:1) (Promega, Madison, WI, USA) using Lipofectamine 2000. The cells were infected with adenoviruses at day 1 and lysate was collected using lysis buffer (Promega) at day 3. Luciferase and renilla activities were examined using dual-luciferase reporter assay kit and a luminometer (Promega). All experiments were performed in triplicate.

### Cell viability assay

Cells were seeded at 1 × 10^3^ cell/well in 96-well plate and infected with the indicated MOI of the adenoviruses. Seven days later, viable cells were quantified using Cell Counting Kit-8 (CCK-8) assay kit (Dojindo Molecular Technologies, Japan) on a microplate reader (Tecan, Switzerland). The assays were performed in triplicate.

### Colonosphere formation assay

To assess sphere formation potential, various transduced cells were seeded into low-attachment 24-well plate at 2 × 10^3^ cells per well. The cells were cultured in stem cell medium for 14 days to obtain colonospheres [21, 29]. Methyl cellulose (1%, Sigma Aldrich) was added to prevent cell aggregation, ensuring individual sphere originated from a single cell. Fresh stem cell medium was replaced every 3 days. More than 10 repeat wells were counted for each group and spheres with a diameter larger than 50 μm were included. To examine sphere formation in serial passages, parental colonospheres were dissociated into single-cell suspension and reseeded. The experiments were performed in triplicate.

### Anchorage-independent colony formation assay

To examine the tumorigenicity of CRC cells *in vitro*, 5 × 10^3^ cells were suspended in 0.7% agar-containing medium and then plated into 6 cm dishes (Costar) pre-coated with 1.2% agar-containing medium. Cells were incubated at 37°C for 2 to 4 weeks and then colonies with a diameter higher than 75 μm were counted. The experiments were repeated for three times.

### Glycolysis assay

Glucose consumption was assessed with Glucose Uptake Colorimetric Assay Kit (Catalog ^#^ K676; Biovision). Briefly, 1.5 × 10^3^ ALDH^+^ CRC cells were infected with adenoviruses and seeded at each well in a 96-well plate. Forty eight hrs later, the cells were starved by pre-incubating with 100 μl Krebs-Ringer-Phosphate-HEPES (KRPH) buffer containing 2% BSA for 40 min, followed by addition of 2-DG and 20 min incubation. Absorbance was measured at 412 nm in a microplate reader and glucose uptake in these cells was determined according to the standard curves.

To quantify the lactate produced by CRC cells, ALDH^+^ cells were transfected and seeded at the density of 5 × 10^5^ cells per well at 6-well plate. The cells were washed with PBS 48 hrs later and incubated with fresh medium. After 2 hrs, aliquots of the culture medium were harvested for analysis of lactic acids using an Accutrend lactate analyzer with a linear range of standard lactate concentrations (Roche, Germany).

PDH activity was tested following the procedures described elsewhere [31].

### Animal experiments

All procedures for animal experiments were approved by the Committee on the Use and Care of Experimental Animals (The Third Military Medical University) and performed in accordance with the institutional guidelines. We performed three types of animal experiments as following.

Limiting dilution assay was performed to calculate the cell dose at which tumor was derived by a single CSC *in vivo*. Briefly, ALDH^+^ HCT116 cells were transduced with adenoviruses and diluted serially to the desired cell doses. The cells were inoculated subcutaneously into the flanks of nude mice. Two months later, the numbers of tumors out of the number of injections were numerated to calculate the frequency of CICs using the ELDA software (http://bioinf.wehi.edu.au/software/elda/index.html) [3, 29]. To examine the self-renewal capacity of CSCs *in vivo*, xenograft tumors were dissected and ALDH^+^ tumor cells were sorted by FACS and re-injected into secondary animals in a limiting dilution manner to assess the frequency of secondary CICs.

To monitor the growth of xenograft tumors, ALDH^+^ HCT116 cells (5 × 10^5^) were subcutaneously inoculated into the right flanks of 4 to 6 week-old BALB/c nude mice (Center for Experimental Animals, Third Military Medical University). When tumors reached between 6 to 9 mm in diameter, the mice were randomly assigned to PBS-, Ad-EGFP, Ad-lnc-p21 and Ad-lnc-p21-MRE groups (six mice per group). The adenoviruses were administrated *i.v*. at 1 × 10^9^ pfu per injection every 4 days for 5 times. Tumor growth was measured periodically with caliper and tumor volume was calculated using the formula: Volume (mm^3^) = length (mm) × width (mm)^2^ / 2. At the end of adenovirus therapy, the xenograft tumors were dissected and weighed. In our experiments, no mice were died of tumor loading.

To further study potential side effect from Ad-lnc-p21 treatment, we used a group of tumor-free naïve mice to study the effect of indicated constructs (Ad-EGFP, Ad-lnc-p21 and Ad-lnc-p21-MRE) on healthy animal (group = 4; *n* = 6). The adenoviruses were injected *i.v*. at 1 × 10^9^ pfu per six-week-old male mice every 4 days for 5 times. Control mice were treated with PBS. The body weights of mice were measured after adenovirus therapy. Any usual signs were recorded during this experiment. Animal blood (2 ml) was harvested by cardiac puncture on Day 20 and incubation with 12 U of heparin. The concentration of Alanine aminotransferase (ALT) in mice serum was measured using ALT Activity Assay Kit (ab105134, Abcam).

### Statistical analysis

The statistical tests in this study were two-tailed student *t*-test. To examine the differences in the frequencies of cancer-initiating cells (CICs), extreme limiting dilution assay (ELDA) software (http://bioinf.wehi.edu.au/software/elda/index.html) were applied. Differences were considered as statistically significant (*) when *P* < 0.05 and statistically very significant (**) when *P* < 0.01.

## SUPPLEMENTARY FIGURES


