# Supplementary material for: miRNA-regulated delivery of lincRNA-p21 suppresses β-catenin signaling and tumorigenicity of colorectal cancer stem cells
Source: Oncotarget. 2015 Oct 16;6(35):37852–70. doi: 10.18632/oncotarget.5635 (PMC4741970; doi:10.18632/oncotarget.5635)
Supplement: Supplementary file 1 [file oncotarget-06-37852-s001.pdf]

SUPPLEMENTARY FIGURES

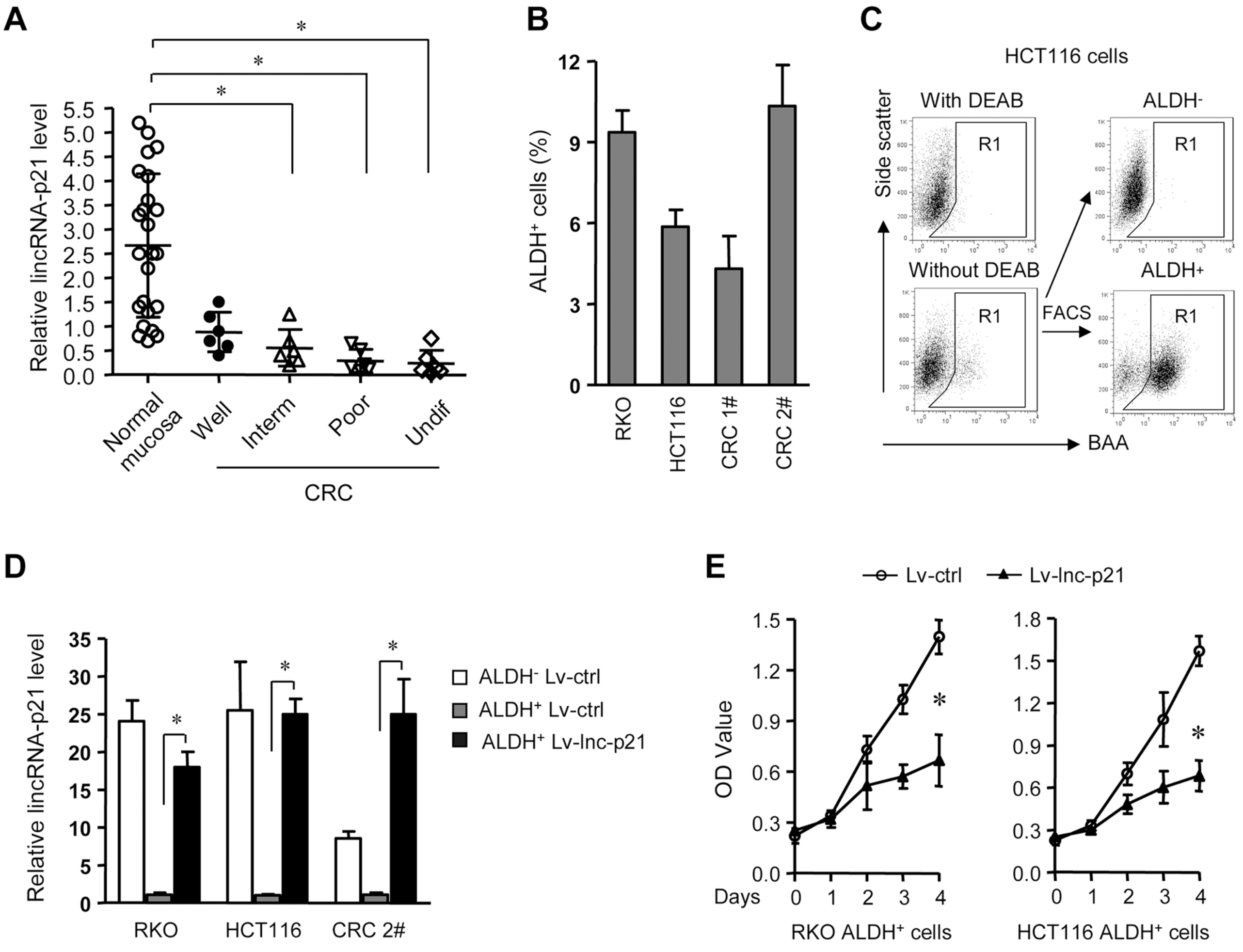

Supplementary Figure S1: LincRNA-p21 suppresses the growth of ALDH⁺ CSCs. A. LincRNA-p21 expression was determined in normal colon mucosa and CRC samples of different grades of differentiation (Inter, intermediate; undif, undifferentiated). **B.** The percentages of ALDH⁺ cells in the colorectal cancer cell lines were analyzed by flow cytometry. **C.** The workflow indicates flow cytometric sorting of ALDH⁺ CSCs and ALDH⁻ non-CSCs from HCT116 cells. **D.** The expression level of lincRNA-p21 was examined by qPCR in control ALDH⁻ cells and ALDH⁺ CSCs infected with Lv-lincRNA-p21 (Lv-lnc-p21) or control vector (Lv-ctrl) at 10 MOI for 48 hrs. **E.** The proliferation of ALDH⁺ CSCs infected with Lv-lnc-p21 or Lv-ctrl was measured using CCK-8 kit. Representative graphs (B) are shown. Data are presented as the mean ± SD (A, B, D, E) of each group from triple replicates. *$P < 0.05$.

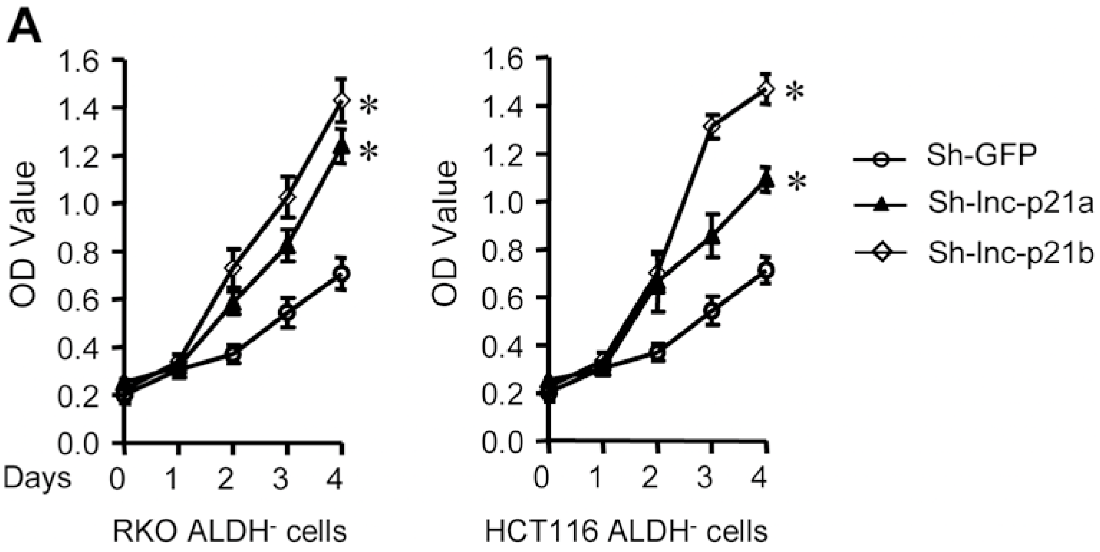

Supplementary Figure S2: Knockdown of lincRNA-p21 in ADLH⁻ cells promotes cell proliferation. A. ALDH⁻ cells were infected with lentivirus expressing Sh-lnc-p21a, Sh-lnc-p21b, or Sh-GFP. The growth rate was measured using CCK-8 kit. Data are presented as the mean ± SD of each group from triple replicates. $^{*}P < 0.05$.

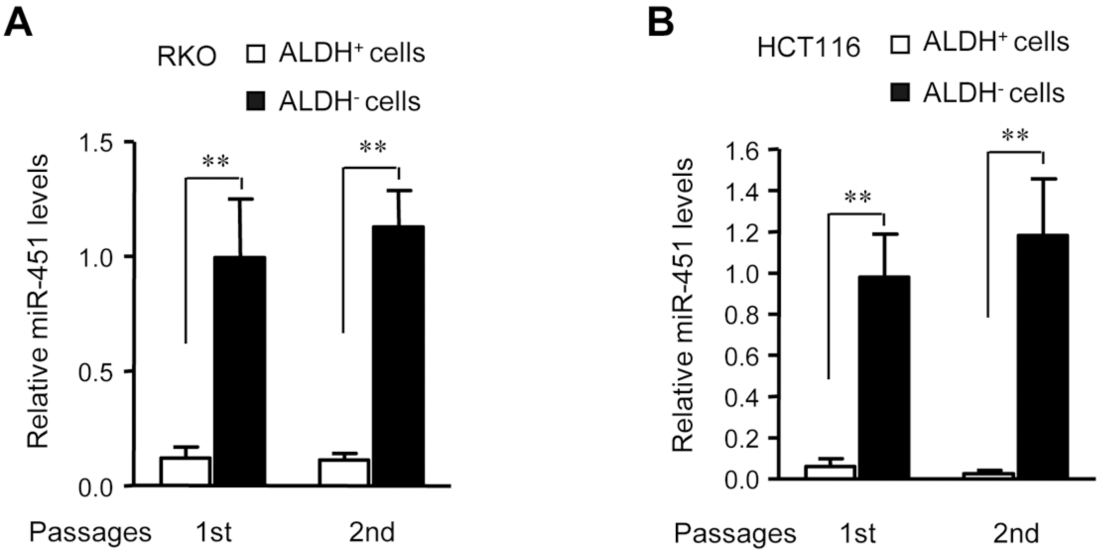

Supplementary Figure S3: Reduced expression of lincRNA-p21 in ALDH⁺ CSCs. A, B. ALDH⁺ or ALDH⁻ subsets of CRC cells were sorted by FACS from RKO cells (A) and HCT116 cells (B). The cells were maintained as colonospheres in stem cell medium. The expression levels of miR-451 in first and second generation of colonospheres derived from ALDH⁺ or ALDH⁻ cells were measured by qPCR. *U6* was used as an endogenous control. Data are presented as the mean ± SD (A, B) of each group from triple replicates. $^{**}P < 0.01$.

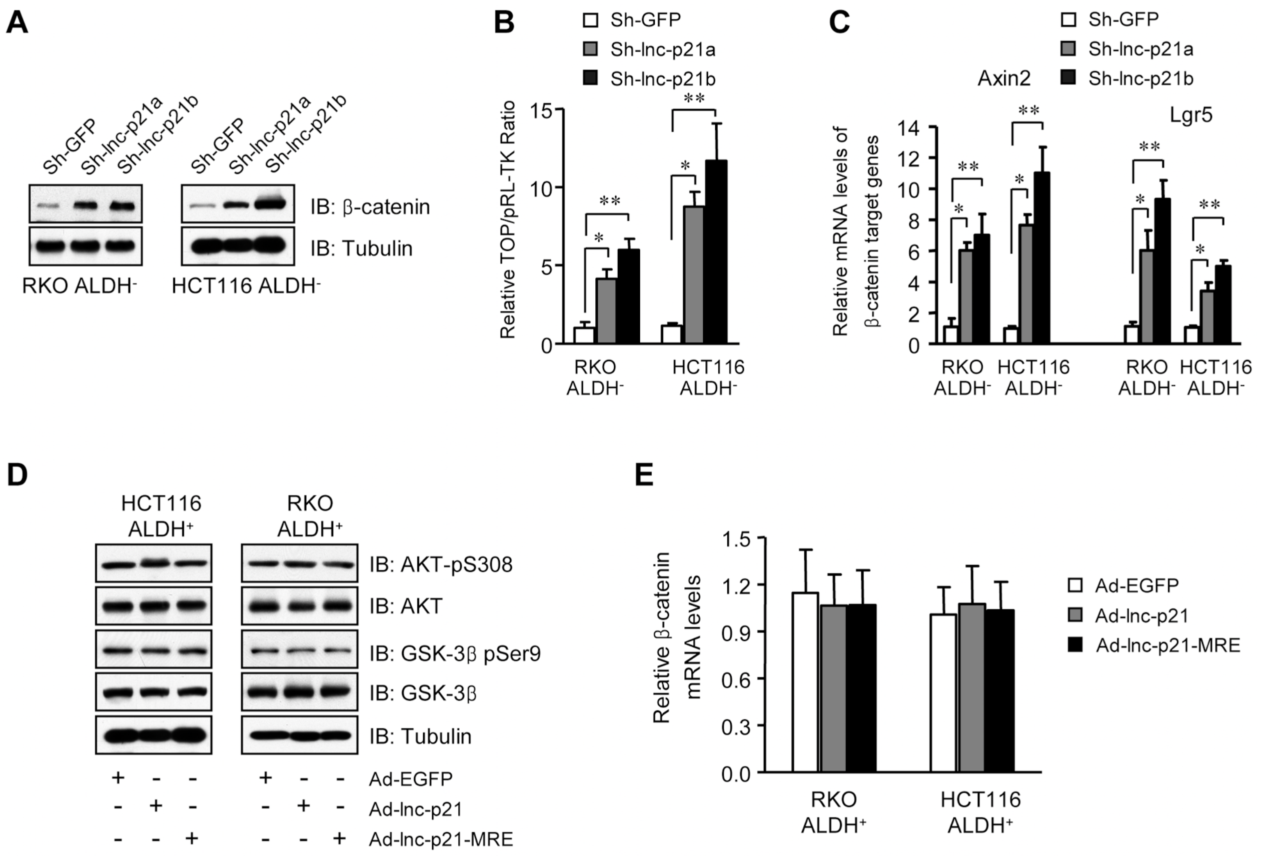

Supplementary Figure S4: Knockdown of lincRNA-p21 elevates β-catenin expression and signaling activity in ADLH⁻ cells. A. Western blot analysis of the levels of β-catenin in ALDH⁻ cells infected with lentivirus expressing Sh-lnc-p21a, Sh-lnc-p21b, or Sh-GFP. Tubulin served as a loading control. **B.** These cells were co-transfected with a mixture of TOPFlash reporter plasmids and pRL-TK control plasmids (1 µg / 1 × 10^6 cells; 40:1) for 48 hrs. TOPFlash reporter activity was examined to quantify the activity of β-catenin signaling. **C.** mRNA levels of β-catenin target genes, Axin2 and Lgr5, were measured by qPCR in these ALDH⁻ cells. GAPDH served as an endogenous control. **D.** ALDH⁺ CSCs were infected with Ad-EGFP, Ad-lincRNA-p21 and Ad-lincRNA-p21-MRE (10 MOI) for 48 hrs. Phosphorylation and protein expression of AKT and GSK-3β were examined by western blot. Tubulin served as a loading control. **E.** qPCR analyses of β-catenin mRNA levels in ALDH⁺ CSCs infected with lincRNA-p21-expressing adenoviruses. Representative images (A, D) are shown. Data are presented as the mean ± SD (B, C, E) of each group from triple replicates. $*P < 0.05$, $**P < 0.01$.

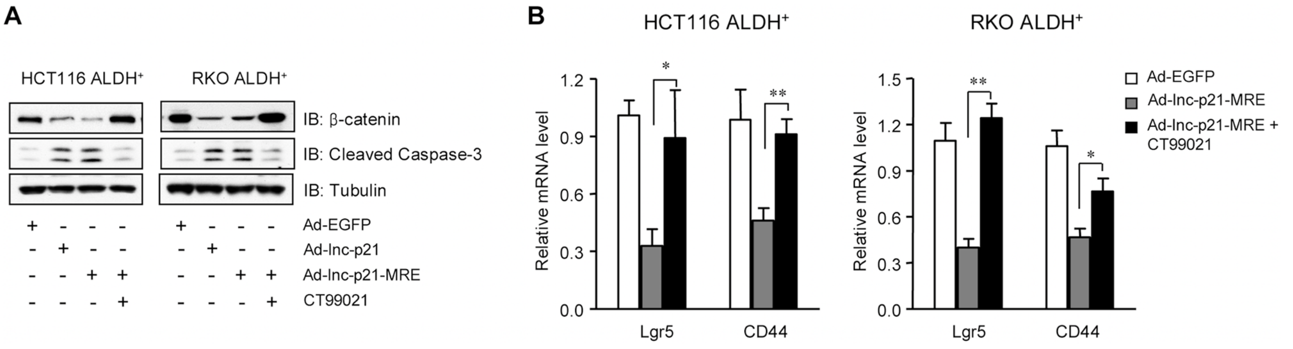

Supplementary Figure S5: Inhibition of GSK-3 by CT99021 restores β-catenin expression and signaling activity in ADLH+ CSCs overexpressing lincRNA-p21. A. ALDH+ cells were infected with Ad-EGFP, Ad-lnc-p21, or Ad-lnc-p21-MRE for 48 hrs. CSCs infected with Ad-lnc-p21-MRE were co-incubated with or without CT99021(3 μM). The levels of β-catenin and cleaved caspase-3 in these cells were detected by western blot analysis. Tubulin served as a loading control. **B.** Total mRNA was collected and transcript levels of β-catenin target genes, CD44 and Lgr5, were measured by qPCR in these cells. GAPDH served as an endogenous control. Representative images (A) are shown. Data are presented as the mean ± SD (B) of each group from triple replicates. *$P < 0.05$, **$P < 0.01$.

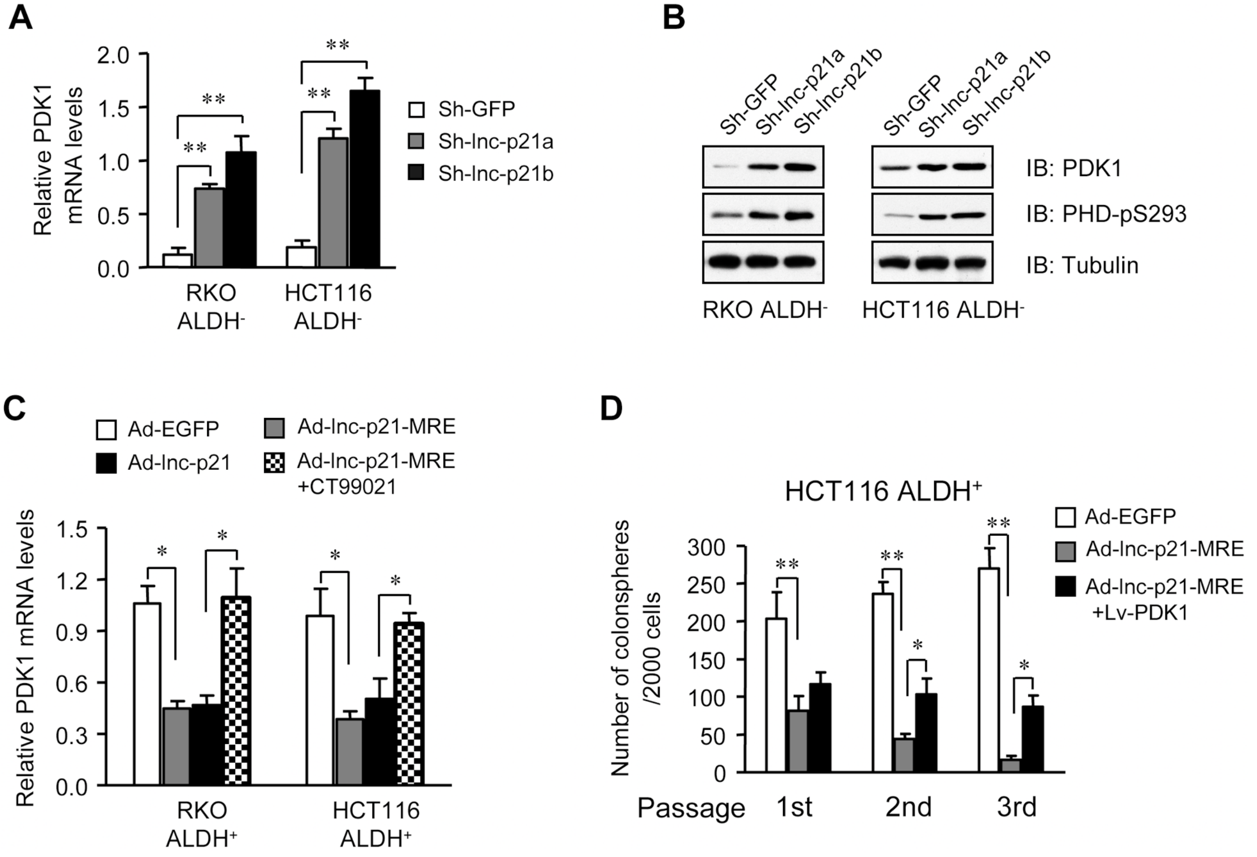

Supplementary Figure S6: Knockdown of lincRNA-p21 upregulates PDK1 expression and PDH phosphorylation. A. ALDH− cells were infected with lentiviruses expressing Sh-lnc-p21a, Sh-lnc-p21b, or Sh-GFP. Transcript levels of PDK1 were measured by qPCR in these cells. GAPDH served as an endogenous control. **B.** The levels of PDK1 and phosphorylated PDH (S293) in these ALDH− cells were examined by western blot. Tubulin was a loading control. **C.** ALDH+ CSCs were treated with Ad-EGFP, Ad-lnc-p21, or Ad-lnc-p21-MRE (10 MOI) for 48 hrs, or Ad-lincRNA-p21-MRE infection together with CT99021 incubation (3 μM). mRNA levels of PDK1 were measured by qPCR, with GAPDH as an endogenous control. **D.** ALDH+ CSCs treated as in (C) were cultured in stem cell medium for 14 days to generate colonospheres in a serial passage manner. The numbers of colonospheres originating from single ALDH+ cells were quantified. Representative images (B) are shown. Data are presented as the mean ± SD (A, C, D) of each group from triple replicates. *$P < 0.05$, **$P < 0.01$.

A

Passages *in vivo*	# of cells	Ad-EGFP	Ad-lnc-p21	Ad-lnc-p21-MRE
1st	10000	7/8	4/8	5/8
	1000	5/8	2/8	2/8
	100	3/8	1/8	1/8
2nd	10000	8/8	4/8	4/8
	1000	6/8	3/8	2/8
	100	3/8	0/8	0/8

B

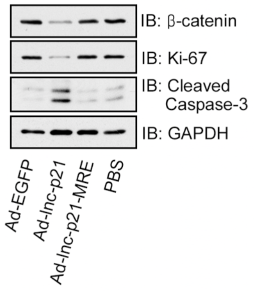

C

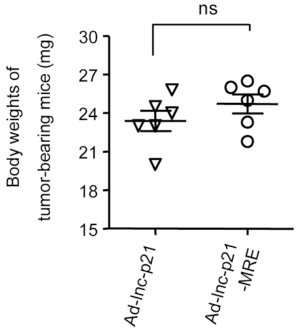

D

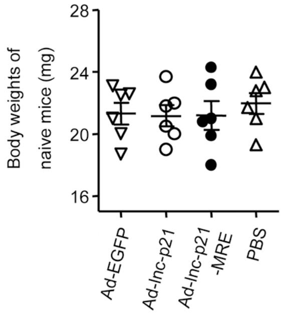

E

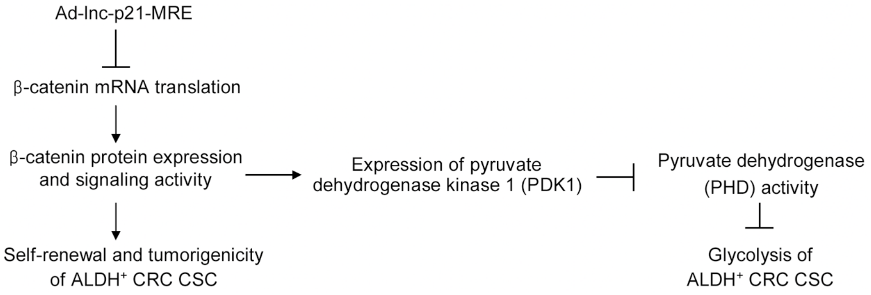

Supplementary Figure S7: The effectiveness and side effects of systemic delivery of Ad-lnc-p21 and Ad-lnc-p21-MRE on recipient mice. A. Tumorigenic incidence of $ALDH^+$ HCT116 cells at indicated cell dosages in nude mice. Serial transplantation assays were also performed in a limiting dilution manner. **B.** Western blot analyses of β-catenin, Ki-67, and cleaved caspase-3 expression in the colon mucosa of recipient mice. GAPDH served as a loading control. **C.** Whole body weights of tumor-bearing mice treated with ether Ad-lnc-p21 or Ad-lnc-p21-MRE. **D.** Body weights of tumor-free naive mice that were administrated *i.v.* with the adenoviruses or PBS. Representativ images (B) are shown. Data are presented as the mean ± SD (C, D) of each group from triple replicate. **E.** A graph illustrating the therapeutic effects and the underlying mechanisms of Ad-lnc-p21-MRE on stemness, glycolysis and tumorigenicity of $ALDH^+$ CSCs.
